# The Relationship Between Different Components and Levels of Physical Exercise, Depressive Symptoms, Inhibitory Control, and Possible Cognitive Neural Mechanisms in College Students

**DOI:** 10.1111/cns.70520

**Published:** 2025-07-31

**Authors:** Shufan Li, Shuqi Jia, Somang Yun, Zhaohui Guo, Xing Wang, QingWen Zhang

**Affiliations:** ^1^ Shanghai University of Sport Shanghai China

**Keywords:** college students, depressive symptoms, ERP, inhibitory control, physical exercise

## Abstract

**Objective:**

Based on event‐related potential (ERP) evidence, this study aims to identify specific indicators of inhibitory control in college students with depressive symptoms, explore the relationship between different components and levels of physical exercise and the specific indicators of depressive symptoms and inhibitory control, and clarify potential targets for exercise interventions and possible mechanisms for alleviating depressive symptoms in college students.

**Methods:**

An observational research design was adopted, utilizing convenience sampling to randomly recruit 225 college students. Participants were asked to complete a demographic questionnaire, the Beck Depression Inventory, and the Physical Activity Scale. Behavioral performance during inhibitory control tasks, as well as synchronous ERP brain signals, was collected.

**Results:**

Compared to healthy college students, the specific indicators of inhibitory control in college students with depressive symptoms included the Nogo accuracy and the N2 amplitude of Fz potentials under the Nogo condition for response inhibition, and the Stroop incongruent reaction time and the P3 amplitude of Fz potentials under the Stroop incongruent condition for interference inhibition (all *p* < 0.05). Physical exercise volume, intensity, duration, and frequency were significantly correlated with depressive symptom scores (all *p* < 0.05). Intensity, duration, and frequency were identified as key influencing factors. Moderate‐intensity sustained exercise and high‐intensity non‐sustained exercise, as well as high‐intensity sustained exercise, showed stronger effects. Longer durations (31–59 min and > 60 min) and higher frequencies (1–2 times/week and 3–5 times/week) also showed stronger effects. Physical exercise volume and intensity were significantly correlated with the N2 amplitude of Fz potentials under the Nogo condition for response inhibition (*p* < 0.05), with no significant differences observed for different exercise intensities (*p* > 0.05). Physical exercise volume, intensity, and duration were significantly correlated with Stroop incongruent reaction time and the P3 amplitude of Fz potentials under the Stroop incongruent condition (all *p* < 0.05). Intensity and duration were key influencing factors. In terms of behavioral task performance, moderate‐intensity sustained exercise and high‐intensity non‐sustained exercise, as well as high‐intensity sustained exercise, exhibited stronger effects. Regarding cognitive neural processing, high‐intensity non‐sustained exercise had a more substantial effect, and durations of 31–59 and > 60 min also had stronger effects.

**Conclusion:**

College students with depressive symptoms exhibit impaired inhibitory control, with decreased behavioral performance in response inhibition and interference inhibition tasks and reduced cognitive neural processing abilities. These can serve as key indicators for the early identification of depressive symptoms in college students. For depressive symptoms, it is recommended that exercise intensity be moderate or higher, with a duration of at least 30 min and a frequency of 1–2 times/week and 3–5 times/week, with the optimal frequency being 3–5 times/week. For interference inhibition, it is recommended that exercise intensity be moderate or higher, with the greatest benefits observed from high‐intensity non‐sustained exercise for cognitive neural processing and a duration of at least 30 min. When designing exercise programs, it is important to consider the combination of different components of exercise and to tailor personalized, precise interventions based on individual differences in depressive symptoms and target areas.

## Graphical abstract

College students with depressive symptoms exhibit decreased behavioral performance in response inhibition and interference inhibition tasks, as well as reduced cognitive neural processing abilities. Depressive symptoms: Moderate/high exercise, ≥ 30 min, 3–5 times/week. Interference inhibition: Moderate/high exercise, high‐intensity non‐sustained exercise best for cognitive neural processing, ≥ 30 min.

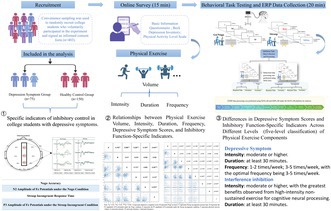

## 1 Introduction

Depression is the leading cause of disability among mental illnesses, severely affecting human life and health. The disability‐adjusted life years (DALYs) due to depression rank second among all diseases and injuries [1]. Depression is characterized by significant and persistent low mood, reduced volition, and cognitive impairments, along with physical symptoms such as appetite loss and insomnia. In severe cases, it may lead to self‐harm or suicidal thoughts and behaviors [2, 3]. In recent years, depression has increasingly affected younger populations. The global point prevalence of self‐reported depressive symptoms is 34%, while among adolescents, it rises to 37% [4]. College students are in a critical period of psychological and physical development, making them a vulnerable group for mental health issues. The detection rate of depressive symptoms in this population is as high as 30%, with a trend of increasing year by year [5, 6]. Therefore, early identification and prevention of depressive symptoms in college students are urgently needed.

Inhibitory control includes response inhibition and interference inhibition. Individuals with depressive symptoms often exhibit impaired inhibitory control, which is closely related to depressive symptoms. Response inhibition refers to the ability to suppress dominant or habitual responses, including the inhibition of impulsive or inappropriate behaviors. Interference inhibition refers to the ability to exclude or reduce distractions from irrelevant information, maintain focus on the primary task, and resolve conflicts caused by distracting information [7]. Studies have found that the higher the depressive symptom score, the poorer the performance on inhibitory control tasks [8]. Individuals with depressive symptoms have difficulty controlling impulses and excluding distractions, becoming immersed in negative thought patterns, which in turn exacerbate negative emotions [9]. Event‐related potentials (ERP) studies have also found specific alterations in brain activity in individuals with depressive symptoms. Compared to the normal control group, they show reduced P3 amplitude and prolonged N2 and P3 latencies [10, 11]. Individuals with depressive symptoms show difficulty in inhibiting negative information in response inhibition tasks [12], with reduced N2 and P3 amplitudes [13, 14].

Physical exercise, inhibitory control, and depressive symptoms are closely related. Physical exercise is an effective means of preventing or alleviating depressive symptoms, offering advantages such as ease of implementation, high adherence, minimal side effects, and stable effects [15, 16, 17]. Cross‐sectional studies have found that a lack of physical exercise is a risk factor for depressive symptoms in college students [18], and regular physical exercise is associated with a reduced risk of depression [19, 20, 21]. A meta‐analysis has found that physical activity at different dosages can reduce the risk of developing depression [22]. Additionally, physical exercise can improve depressive symptoms by enhancing inhibitory control. The possible mechanism is that exercise can remodel the brain structure in individuals with depression, activate the function of related brain regions, and maintain the integrity of hippocampal and white matter volumes, thus improving brain neural processing ability and promoting behavioral adaptive changes [23]. Existing studies have found that exercise induces positive changes in ERP (N2 and P3 amplitude increases) related to inhibitory control in individuals with depression. Both single and long‐term exercise can improve inhibitory control and thus alleviate depressive symptoms [24, 25, 26].

A review of previous studies reveals that individuals with depressive symptoms exhibit impaired inhibitory control, and there is a close relationship between physical exercise, depressive symptoms, and inhibitory control. However, to date, the specific indicators of inhibitory control in college students with depressive symptoms remain unclear. It is uncertain whether response inhibition or interference inhibition is impaired, and further clarification is needed regarding task performance and cognitive neural processing mechanisms related to the impaired dimensions. Whether there is a relationship between the physical exercise volume, intensity, duration per session, frequency of exercise, depressive symptom scores, and specific inhibitory control indicators requires further evidence to confirm. If such relationships exist, how the differences in depressive symptom scores and specific inhibitory control indicators are affected by various exercise components (intensity, duration, frequency) and levels (five‐level classification) still need to be explored. Based on this, the present study uses an observational research design to address the above research questions, clarify the specific indicators of inhibitory control in college students with depressive symptoms, and explore targeted exercise interventions for these students. The goal is to provide a reference for early identification of depressive symptoms in college students, contribute to the prevention and intervention of depressive symptoms, and provide a theoretical basis for developing precise exercise programs in clinical settings.

## 2 Research Subjects and Methods

### 2.1 Research Subjects

Sample Size Estimation: This study used an observational research design and conducted sample size estimation using G*Power 3.1 software. The study includes multiple statistical analyses, with different methods for sample size estimation, as described below: (1) To explore the differences in inhibitory control between college students with depressive symptoms and healthy college students, a t‐test was chosen, with the statistical test option “Means: Difference between two independent means (two groups).” The effect size was set to a medium effect (0.5) [27], *α* was set at 0.05, and the power of the test was set to 0.9. The required sample size per group was 70 participants. Considering a 10% attrition rate, 77 participants were planned to be recruited for the depressive symptom group. The ratio of depressive symptom group to healthy control group was set at 1:2, with 154 participants planned for the control group. (2) To explore the relationship between physical exercise, depressive symptoms, and inhibitory function, an Exact test was chosen with the statistical test option “Correlation: Bivariate normal model” and “Two” tails. The effect size was set to a medium effect (0.3) [27], *α* was set at 0.05, and the power of the test was set to 0.9. The required total sample size was 112 participants. Considering a 10% attrition rate, 123 participants were planned to be recruited. All statistical analyses in this study were conducted on the same group of participants; therefore, it is necessary to ensure that the sample size meets the requirements for all analyses, with the maximum number being 77 participants in the depressive symptom group and 154 participants in the healthy control group.

Inclusion criteria: Enrolled college students aged 18–22 years; no major organic diseases; no history of psychiatric disorders and no use of psychotropic medications; no chronic diseases; no contraindications to exercise; normal or corrected vision, no color blindness or color weakness; right‐handed; Beck Depression Inventory (BDI) score of 14–28 for the depressive symptom group; BDI score < 14 for the healthy control group; voluntary participation in this study. Exclusion criteria: Participants who withdraw from the experiment; participants who drop out; excessive EEG artifacts, resulting in unanalyzable data; data loss.

Sampling: Convenient sampling method was used to randomly recruit college students who voluntarily participated in the experiment at a university in Shanghai, through health education seminars, posting participant recruitment posters, and other methods. Participants were informed of the study's purpose and content and signed an informed consent form.

**FIGURE 1:**
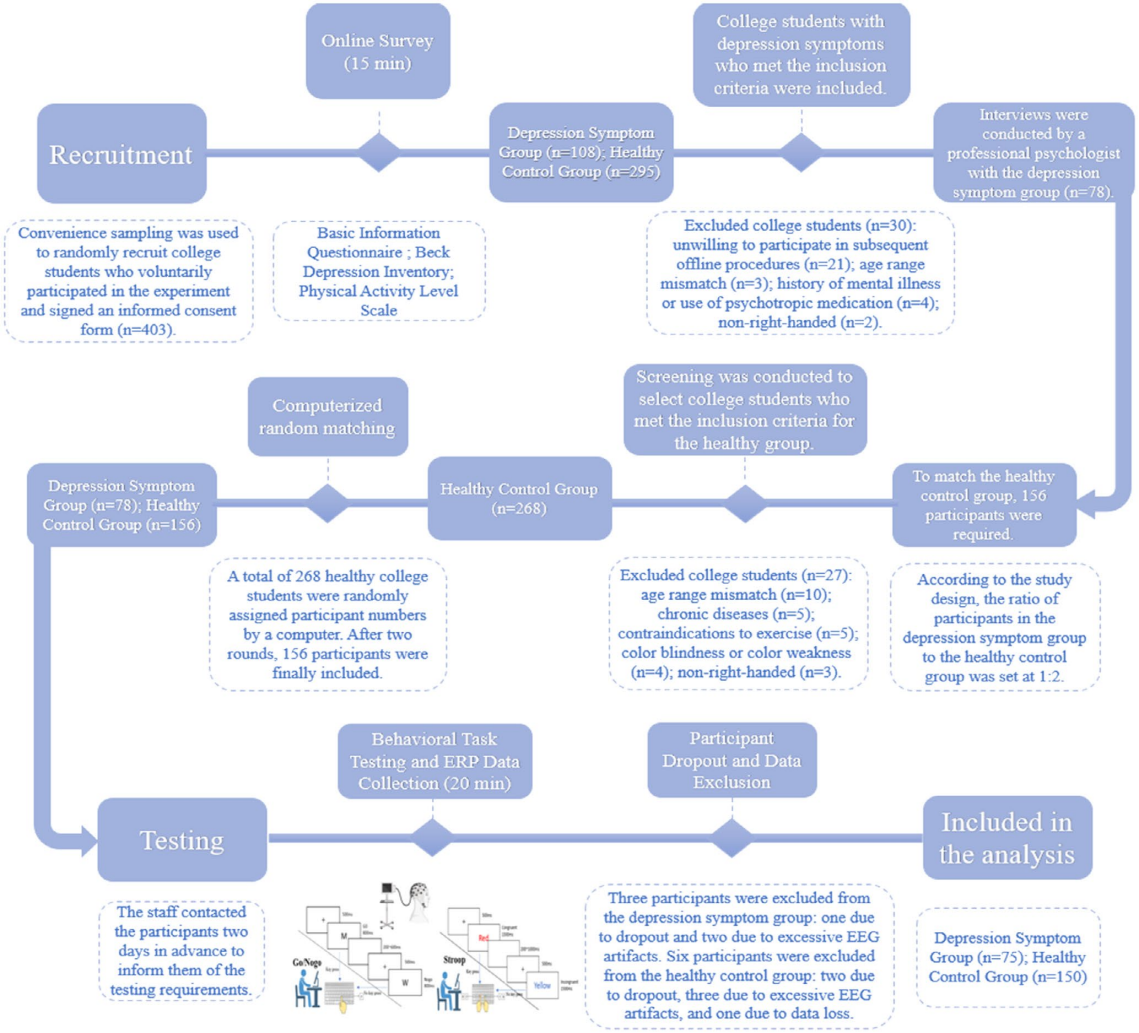
Recruitment and testing procedure.

Recruitment and testing procedure: This study consisted of three stages. In the first stage, participants completed an online questionnaire, including a demographic questionnaire, the BDI, and the Physical Activity Level Scale. A total of 403 college students were recruited, of which 108 had depressive symptoms and 295 were healthy controls. Based on voluntary participation, eligible participants with depressive symptoms were invited via phone for offline interviews, during which professional psychological counselors conducted face‐to‐face consultations. A total of 78 students with depressive symptoms participated in the study. In the second stage, according to the study design, the ratio of the depressive symptom group to the healthy control group was set at 1:2, with the healthy control group requiring 156 participants. Among the normal college students, 268 met the inclusion criteria. A random selection of 156 participant IDs was generated by computer, and participants were invited by phone. A total of 136 agreed to participate. To compensate for missing participants, 20 more participant IDs were randomly generated from the remaining 112, and these were invited by phone. Of these, 20 agreed to participate, bringing the final total to 156 healthy college students who participated in the subsequent research. The third stage involved task testing. Participants were contacted 2 days in advance and instructed to avoid strenuous exercise in the 24 h before testing, maintain adequate sleep, follow a normal diet, and refrain from consuming alcohol, coffee, or other stimulants within 12 h prior to testing. They were also advised to wash their scalp before arriving at the testing site. Upon arrival at the testing site, participants were briefed on the test procedure, signed an informed consent form, and completed a 20‐min inhibitory control task along with ERP brain signal collection. The recruitment and testing procedure is shown in Figure 1.

This study complies with the ethical requirements of the latest version of the Declaration of Helsinki and has been approved by the Ethics Committee of Shanghai University of Sport (102772023RT075).

### 2.2 Testing Tools

#### 2.2.1 Basic Information Questionnaire

The content of the basic information questionnaire includes gender, age, height, weight, handedness, vision status, hearing status, medical history, and medication use. Body mass index (BMI) is calculated by dividing weight (kg) by the square of height (m), with the formula BMI = weight (kg)/height^2^ (m).

#### 2.2.2 Beck Depression Inventory‐II (BDI‐II)

The BDI‐II is one of the most widely used self‐report scales for depressive symptoms, with an internal consistency coefficient of 0.948 [28]. The scale contains 21 items, scored on a 0–3 point scale, with a total score of 63. A total score of 0–13 indicates no depression, 14–19 indicates mild depression, 20–28 indicates moderate depression and 29–63 indicates severe depression.

#### 2.2.3 The Physical Activity Rating Scale‐3 (PARS‐3)

The PARS‐3 is based on the Chinese version revised by Liang Deqing et al. The internal consistency reliability of the scale ranges from 0.80 to 0.86, and the test–retest reliability is 0.82 [29]. This scale primarily reflects an individual's physical activity over the past month. It includes intensity, duration of each exercise session, and frequency, with intensity and frequency scored on a 1–5 scale, and duration scored on a 0–4 scale. The physical exercise volume is calculated as: intensity × duration × frequency, with a score range of 0 to 100.

#### 2.2.4 Inhibition Function Task and ERP Data Collection and Processing

##### 2.2.4.1 Response Inhibition Task

**FIGURE 2:**
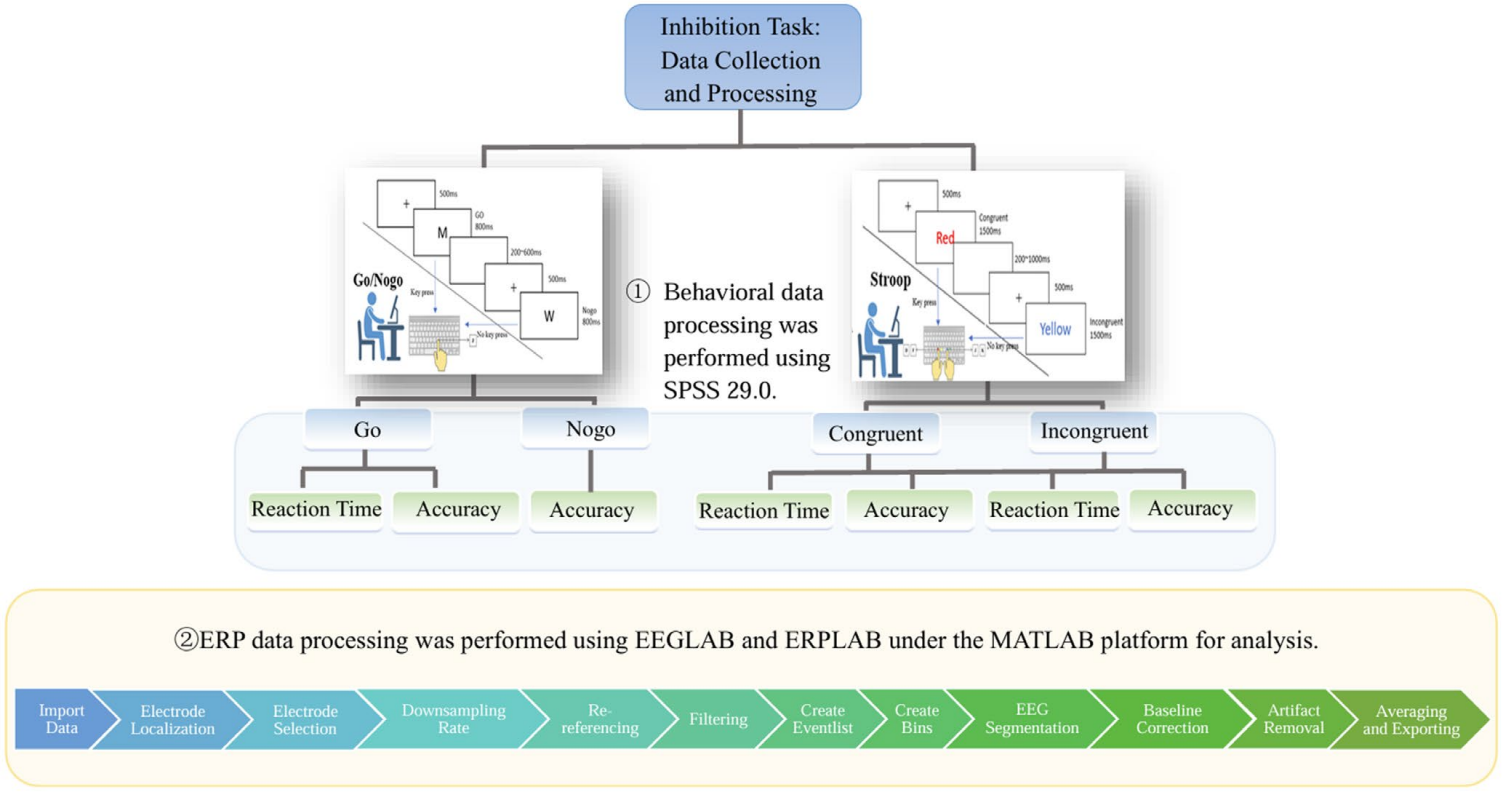
Data collection and processing flow for the inhibition function task.

The Go/Nogo task is used to measure participants' response inhibition ability, implemented using MATLAB 2022b (Figure 2). The stimuli are presented randomly, with “M” representing the Go stimulus and “W” representing the Nogo stimulus. When “M” appears, participants are required to press the “J” key quickly and accurately, while no key press should occur when “W” is presented. After pressing the key, the target stimulus immediately disappears, and if no key is pressed, the target stimulus disappears automatically after 800 ms. Prior to the formal experiment, there are 20 practice trials for participants to become familiar with the procedure. After familiarization, they proceed to the formal experiment. During the formal experiment, a fixation point is presented at the center of the screen for 500 ms, followed by random stimulus presentation, each stimulus lasting 800 ms, with a random inter‐stimulus interval of 200–600 ms. The task consists of 120 trials, with 90 Go trials and 30 Nogo trials [30]. After the task, the computer records the participants' accuracy and reaction time.

##### 2.2.4.2 Interference Inhibition Task

The Stroop task is used to measure participants' interference inhibition ability, implemented using MATLAB 2022b (Figure 2). The stimuli are presented randomly, with the Chinese characters “红(Red) “,”黄(Yellow)”,”蓝(Blue) “, and “绿(Green)” displayed in red, yellow, blue, and green colors, respectively. A total of 16 stimulus materials are used, including 4 congruent materials (color word matches the color) and 12 incongruent materials (color word does not match the color). Participants are required to ignore the meaning of the word and judge its color, pressing the key corresponding to the color as quickly and accurately as possible: “D” for red, “F” for yellow, “J” for blue, and “K” for green. After pressing the key, the target stimulus immediately disappears; if no key is pressed, the stimulus disappears automatically after 1500 ms. Prior to the formal experiment, there are 20 practice trials for participants to become familiar with the procedure. Once familiarized, they proceed to the formal experiment. During the formal experiment, a fixation point is shown for 500 ms, with each stimulus displayed for 1500 ms and a random inter‐stimulus interval between 200 and 1000 ms. The task consists of 72 trials, with 36 congruent trials and 36 incongruent trials. After the task, the computer records the participants' accuracy and reaction time.

##### 2.2.4.3 ERP Collection and Processing

ERP are collected while participants perform behavioral tasks, with EEG signals recorded simultaneously. Data analysis is conducted using EEGLAB and ERPLAB on the MATLAB platform. The main steps in the analysis include: importing data; electrode localization; selecting electrodes; setting the sampling rate to 256 Hz; re‐referencing to electrodes A1 and A2; applying a band‐pass filter between 0.1 and 30 Hz; creating an event list; creating bins; and baseline correction. In ERP studies, EEG signals are segmented according to different task conditions. In this study, data segmentation is carried out based on the method proposed by Mark, extracting key EEG signal segments. The stimulus presentation time is set as 0, with baseline correction applied to the 200 ms prior to the stimulus. Segmentation windows are selected for the Go/Nogo task (−200 to 800 ms) and the Stroop task (−200 to 1000 ms). Independent Component Analysis (ICA) is used to remove artifacts. Signal averaging and exportation are performed to improve the signal‐to‐noise ratio (SNR), and ERP signals induced by the behavioral tasks are exported for individual participants. For batch data, signal averaging is performed within each group (depressed and non‐depressed groups) to compute the overall mean ERP for each group. This study focuses on analyzing Fz, Cz and Pz electrode potentials, based on previous research and ERP data obtained in this study [31, 32]. The N2 component time window for the Go/Nogo task is set between 250 and 400 ms, and the P3 component time window is from 300 to 542 ms. For the Stroop task, the N2 time window is between 170 and 230 ms, and the P3 window is from 220 to 400 ms. The amplitudes and latencies of the N2 and P3 components are averaged based on their respective time windows.

### 2.3 Statistical Methods

Data were statistically analyzed and visualized using SPSS 29.0, JASP 0.17.1, MATLAB 2022b, and R 4.2.3 software. The Kolmogorov–Smirnov test was used to assess normality, supplemented by Q–Q plots and histograms for evaluation. Normally distributed continuous data were expressed as mean ± standard deviation, with results presented to three decimal places. Independent‐samples *t*‐test was used for group comparisons. Categorical data were described using frequencies (*n*), with group comparisons performed using the chi‐square (*χ* ^2^) test. Repeated‐measures analysis of variance (ANOVA) was used to examine differences in ERP results between college students with depressive symptoms and healthy controls. Mauchly's test of sphericity was used to assess the homogeneity of variance, and for data failing the sphericity assumption, Greenhouse–Geisser correction was applied to adjust the degrees of freedom and *p*‐values. Pearson correlation analysis was employed to investigate the associations between measures of inhibitory control differences and depressive symptom scores among college students with depressive symptoms compared to healthy controls. Multiple linear regression analysis was conducted to examine the explanatory power of inhibitory control indicators on depressive symptoms, with depressive symptom scores as the dependent variable and inhibitory control indicators as independent variables. Stepwise regression was used to select significant variables and build the optimal regression model. Pearson correlation analysis was used to investigate the relationship between total physical exercise and depressive symptom scores as well as specific inhibitory control indicators. Spearman correlation analysis was used to explore the relationship between different components of physical exercise and depressive symptom scores and specific inhibitory control indicators. One‐way analysis of variance (ANOVA) followed by post hoc multiple comparisons was used to compare depressive symptom scores and specific inhibitory control indicators across different levels of physical exercise components in college students. All statistical inferences were conducted using two‐tailed tests, with a significance level (*α*) set at 0.05.

## 3 Results and Analysis

### 3.1 Demographic Information of Participants

A total of 225 college students participated in the study (Table 1), including 75 students with depressive symptoms and 150 healthy control group students. No significant differences in demographic data were found between the students with depressive symptoms and the healthy control group (*p* > 0.05 for all comparisons).

**TABLE 1:** Demographic information of college students with different depression symptom scores (*n* = 225).

Variables	College students with different depression symptom scores		Difference test
	Depressive symptom group (*n* = 75)	Healthy control group (*n* = 150)	
Age (years)	19.600 ± 1.053	19.560 ± 1.184	*t* = −0.248, *p* = 0.805
Height (m)	1.675 ± 0.080	1.678 ± 0.086	*t* = 0.257, *p* = 0.798
Weight (kg)	59.632 ± 10.648	60.289 ± 12.222	*t* = 0.396, *p* = 0.692
BMI (kg/m^2^)	21.218 ± 3.378	21.323 ± 3.072	*t* = 0.235, *p* = 0.815
Gender (*n*)			*χ* ^2^ = 0.155, *p* = 0.694
Male	28	52	
Female	47	98	
Place of household registration (*n*)			*χ* ^2^ = 0.266, *p* = 0.606
Urban	51	107	
Rural	24	43	
Only child (*n*)			*χ* ^2^ = 0.009, *p* = 0.925
Yes	39	79	
No	36	71	
Single‐parent family (*n*)			*χ* ^2^ = 1.440, *p* = 0.230
Yes	11	14	
No	64	136	

### 3.2 Inhibitory Control Characteristics in College Students With Depressive Symptoms

#### 3.2.1 Differences in Performance Between Students With Depressive Symptoms and Healthy Control Group Students in Inhibitory Control Tasks

As shown in Table 2, there were significant differences between the depression symptom group and the healthy control group in Nogo accuracy during the response inhibition task; in the interference inhibition task, significant differences were observed in Stroop incongruent accuracy, Stroop congruent reaction time, and Stroop incongruent reaction time (all *p* < 0.05). These results suggest that, compared to healthy college students, depression symptom college students showed declines in Nogo accuracy, Stroop incongruent accuracy, Stroop congruent reaction time, and Stroop incongruent reaction time.

**TABLE 2:** Differences in inhibitory control between students with depressive symptoms and healthy control group students (*n* = 225).

Task	Depressive symptom group (*n* = 75)	Healthy control group (*n* = 150)	Difference test
Response inhibition			
Go accuracy	94.841 ± 5.930	96.133 ± 5.327	*t* = 1.650, *p* = 0.100
Go reaction time	0.326 ± 0.040	0.319 ± 0.037	*t* = −1.384, *p* = 0.168
Nogo accuracy	63.649 ± 14.848	68.997 ± 12.798	*t* = 2.799, *p* = 0.006
Interference inhibition			
Stroop congruent accuracy	98.259 ± 6.308	99.531 ± 1.724	*t* = 1.715, *p* = 0.090
Stroop incongruent accuracy	90.402 ± 10.196	94.183 ± 6.448	*t* = 2.932, *p* = 0.004
Stroop congruent reaction time	0.625 ± 0.098	0.584 ± 0.071	*t* = −3.236, *p* = 0.002
Stroop incongruent reaction time	0.734 ± 0.121	0.668 ± 0.093	*t* = −4.154, *p* < 0.001

#### 3.2.2 Differences in ERP Between Depressive Symptom Group and Healthy Control Group During the Completion of Inhibition Tasks

##### 3.2.2.1 N2 Component of Response Inhibition

**FIGURE 3:**
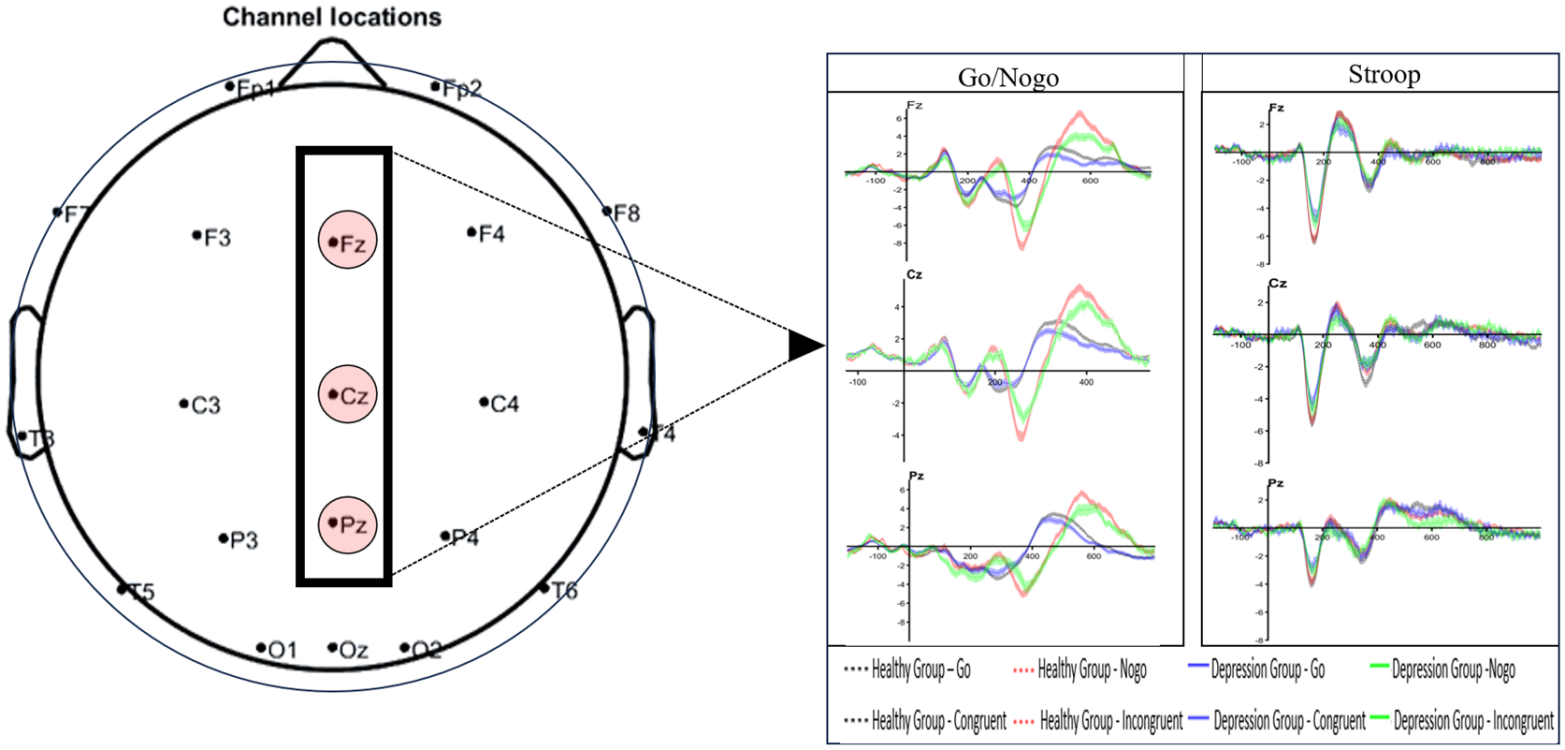
Comparison of N2 and P3 components in inhibitory function tasks between depressive symptom students and healthy control students (*n* = 225).

A 2 (Group) × 2 (Condition) × 3 (Electrode) repeated measures ANOVA was conducted on the N2 amplitude in the Go/Nogo task. The results (Table 3, Figure 3) showed that the main effect of Group was not significant, *F*(1, 223) = 3.814, *p* = 0.052, *ƞp* ^2^ = 0.017; the main effect of Condition was significant, *F*(1, 223) = 179.684, *p* < 0.001, *ƞp* ^2^ = 0.446; the main effect of Electrode was significant, *F*(1.879, 223) = 14.267, *p* < 0.001, *ƞp* ^2^ = 0.060; the Group × Condition interaction was not significant, *F*(1, 223) = 2.247, *p* = 0.135, *ƞp* ^2^ = 0.010; the Group × Electrode interaction was significant, *F*(1.879, 223) = 6.682, *p* = 0.002, *ƞp* ^2^ = 0.029; the Condition × Electrode interaction was significant, *F*(1.976, 223) = 14.189, *p* < 0.001, *ƞp* ^2^ = 0.060; the Group × Condition × Electrode interaction was not significant, *F*(1.976, 223) = 2.267, *p* = 0.106, *ƞp* ^2^ = 0.010. Simple effects analysis revealed that at the Fz electrode, the N2 amplitude of the Healthy Control Group was significantly larger than that of the Depressive Symptom Group. In the Healthy Control Group, significant differences in N2 amplitude were observed across electrodes, with Pz < Cz < Fz. At the Fz, Cz, and Pz electrodes, the N2 amplitude induced by the Nogo condition was significantly larger than that induced by the Go condition. Under the Nogo condition, the N2 amplitude at the Fz electrode was significantly larger than at the Pz electrode, and the N2 amplitude at Cz was significantly larger than at Pz, with Pz < Cz < Fz. These results suggest that, in the Go/Nogo task, the N2 amplitude at the Fz electrode was larger in the Healthy Control Group than in the Depressive Symptom Group.

**TABLE 3:** Comparison of N2 and P3 amplitude and latency in inhibitory function between depressive symptom group and healthy control group (*n* = 225).

Task‐ERP components	Electrode	Depressive symptom group (*n* = 75)		Healthy control group (*n* = 150)	
		Amplitude (μV)	Latency (ms)	Amplitude (μV)	Latency (ms)
Go‐N2	Fz	−5.136 ± 2.989	309.375 ± 53.348	−6.194 ± 2.816	309.609 ± 60.509
	Cz	−5.438 ± 3.105	299.896 ± 57.225	−5.752 ± 2.594	297.318 ± 59.294
	Pz	−5.296 ± 2.988	280.156 ± 51.131	−5.496 ± 2.259	287.240 ± 47.717
Nogo‐N2	Fz	−8.639 ± 5.542	329.115 ± 70.187	−10.863 ± 6.076	341.198 ± 59.226
	Cz	−8.797 ± 4.770	312.917 ± 70.860	−10.220 ± 5.899	333.125 ± 63.256
	Pz	−7.974 ± 5.160	305.052 ± 72.502	−8.300 ± 4.520	321.094 ± 61.717
Go‐P3	Fz	3.689 ± 2.649	457.187 ± 62.128	4.286 ± 2.663	471.536 ± 49.678
	Cz	3.703 ± 2.340	460.729 ± 57.711	4.501 ± 2.458	475.911 ± 50.870
	Pz	4.469 ± 2.456	450.833 ± 60.808	4.803 ± 2.487	470.703 ± 45.111
Nogo‐P3	Fz	5.967 ± 3.783	450.729 ± 88.512	7.627 ± 4.580	447.500 ± 88.957
	Cz	6.030 ± 4.568	459.844 ± 85.956	7.301 ± 5.013	455.599 ± 87.813
	Pz	5.103 ± 4.613	455.052 ± 85.334	5.930 ± 3.764	462.812 ± 82.562
Congruent‐N2	Fz	−5.558 ± 3.793	187.240 ± 29.661	−6.440 ± 3.895	189.063 ± 26.381
	Cz	−4.357 ± 3.451	195.156 ± 30.955	−5.271 ± 3.650	190.651 ± 27.882
	Pz	−3.843 ± 3.303	196.563 ± 36.408	−4.647 ± 3.282	192.292 ± 27.648
Incongruent‐N2	Fz	−5.794 ± 3.994	188.073 ± 28.817	−6.526 ± 3.765	185.547 ± 20.065
	Cz	−5.229 ± 3.319	190.573 ± 29.283	−5.237 ± 3.876	189.714 ± 23.652
	Pz	−4.400 ± 3.309	193.594 ± 35.234	−4.149 ± 3.096	192.578 ± 23.537
Congruent‐P3	Fz	4.334 ± 3.355	270.885 ± 33.887	5.137 ± 3.047	277.214 ± 34.187
	Cz	3.483 ± 3.580	285.833 ± 52.590	3.654 ± 2.709	273.568 ± 42.043
	Pz	3.077 ± 3.035	298.437 ± 56.498	2.765 ± 3.096	286.771 ± 52.492
Incongruent‐P3	Fz	4.516 ± 2.747	278.021 ± 40.556	5.435 ± 3.149	279.036 ± 40.558
	Cz	3.379 ± 2.674	282.604 ± 46.466	4.118 ± 2.512	276.432 ± 44.686
	Pz	2.756 ± 2.574	299.688 ± 57.091	3.587 ± 3.104	290.156 ± 58.167

A 2 (Group) × 2 (Condition) × 3 (Electrode) repeated measures ANOVA was conducted on the N2 latency in the Go/Nogo task. The results (Table 3, Figure 3) showed that the main effect of Group was not significant, *F*(1, 223) = 2.431, *p* = 0.120, *ƞp* ^2^ = 0.011; the main effect of Condition was significant, *F*(1, 223) = 47.237, *p* < 0.010, *ƞp* ^2^ = 0.175; the main effect of Electrode was significant, *F*(1.935, 223) = 26.851, *p* < 0.001, *ƞp* ^2^ = 0.107; the Group × Condition interaction was not significant, *F*(1, 223) = 3.555, *p* = 0.061, *ƞp* ^2^ = 0.016; the Group × Electrode interaction was not significant, *F*(1.935, 223) = 0.342, *p* = 0.703, *ƞp* ^2^ = 0.002; the Condition × Electrode interaction was not significant, *F*(1.930, 223) = 0.387, *p* = 0.672, *ƞp* ^2^ = 0.002; the Group × Condition × Electrode interaction was not significant, *F*(1.930, 223) = 0.773, *p* = 0.458, *ƞp* ^2^ = 0.003. These results suggest that there is no difference in N2 latency between the Depressive Symptom Group and the Healthy Control Group in the Go/Nogo task.

##### 3.2.2.2 P3 Component of Response Inhibition

In the Go/Nogo task, a 2 (Group) × 2 (Condition) × 3 (Electrode) repeated measures ANOVA was conducted with P3 amplitude as the dependent variable. The results (Table 3, Figure 3) showed that the main effect of Group was significant, *F*(1, 223) = 6.749, *p* = 0.010, *ƞp* ^2^ = 0.029; the main effect of Condition was significant, *F*(1, 223) = 71.226, *p* < 0.001, *ƞp* ^2^ = 0.242; the main effect of Electrode was not significant, *F*(1.910, 223) = 1.943, *p* = 0.147, *ƞp* ^2^ = 0.009; the Group × Condition interaction was not significant, *F*(1, 223) = 1.875, *p* = 0.172, *ƞp* ^2^ = 0.008; the Group × Electrode interaction was not significant, *F*(1.910, 223) = 1.286, *p* = 0.277, *ƞp* ^2^ = 0.006; the Condition × Electrode interaction was not significant, *F*(1.844, 223) = 23.829, *p* < 0.001, *ƞp* ^2^ = 0.097; the Group × Condition × Electrode interaction was not significant, *F*(1.844, 223) = 0.610, *p* = 0.531, *ƞp* ^2^ = 0.003. Simple effects analysis revealed that at the Fz, Cz, and Pz electrodes, the N2 amplitude under the Nogo condition was significantly larger than under the Go condition. Under both Go and Nogo conditions, the N2 amplitude at Fz was significantly larger than at Pz, and at Cz, the N2 amplitude was significantly larger than at Pz, with Pz<Cz < Fz. These results suggest that there is no significant difference in P3 amplitude between the Depressive Symptom Group and the Healthy Control Group in the Go/Nogo task.

In the Go/Nogo task, a 2 (Group) × 2 (Condition) × 3 (Electrode) repeated measures ANOVA was conducted with P3 latency as the dependent variable. The results (Table 3, Figure 3) showed that the main effect of Group was not significant, *F*(1, 223) = 1.788, *p* = 0.183, *ƞp* ^2^ = 0.008; the main effect of Condition was not significant, *F*(1, 223) = 2.785, *p* = 0.097, *ƞp* ^2^ = 0.012; the main effect of Electrode was not significant, *F*(1.901, 223) = 1.469, *p* = 0.232, *ƞp* ^2^ = 0.007; the Group × Condition interaction was not significant, *F*(1, 223) = 2.192, *p* = 0.140, *ƞp* ^2^ = 0.010; the Group × Electrode interaction was not significant, *F*(1.901, 223) = 0.855, *p* = 0.421, *ƞp* ^2^ = 0.004; the Condition × Electrode interaction was not significant, *F*(1.945, 223) = 1.948, *p* = 0.145, *ƞp* ^2^ = 0.009; the Group × Condition × Electrode interaction was not significant, *F*(1.945, 223) = 0.152, *p* = 0.853, *ƞp* ^2^ = 0.001. These results suggest that there is no significant difference in P3 latency between the Depressive Symptom Group and the Healthy Control Group in the Go/Nogo task.

##### 3.2.2.3 N2 Component of Interference Inhibition

In the Stroop task, a 2 (Group) × 2 (Condition) × 3 (Electrode) repeated measures ANOVA was conducted with N2 amplitude as the dependent variable. The results (Table 3, Figure 3) showed that the main effect of Group was not significant, *F*(1, 223) = 1.458, *p* = 0.228, *ƞp* ^2^ = 0.006; the main effect of Condition was not significant, *F*(1, 223) = 2.148, *p* = 0.144, *ƞp* ^2^ = 0.010; the main effect of Electrode was significant, *F*(1.849, 223) = 54.723, *p* < 0.001, *ƞp* ^2^ = 0.197; the Group × Condition interaction was significant, *F*(1, 223) = 6.429, *p* = 0.012, *ƞp* ^2^ = 0.028; the Group × Electrode interaction was not significant, *F*(1.849, 223) = 1.190, *p* = 0.303, *ƞp* ^2^ = 0.005; the Condition × Electrode interaction was not significant, *F*(1.999, 223) = 1.699, *p* = 0.184, *ƞp* ^2^ = 0.008; the Group × Condition × Electrode interaction was not significant, *F*(1.999, 223) = 2.564, *p* = 0.078, *ƞp* ^2^ = 0.011. Simple effects analysis revealed that the N2 amplitude induced under the incongruent condition was significantly greater in the Depressive Symptom Group than under the congruent condition. These results suggest that there is no significant difference in N2 amplitude between the Depressive Symptom Group and the Healthy Control Group in the Stroop task.

In the Stroop task, a 2 (Group) × 2 (Condition) × 3 (Electrode) repeated measures ANOVA was conducted with N2 latency as the dependent variable. The results (Table 3, Figure 3) showed that the main effect of Group was not significant, *F*(1, 223) = 0.354, *p* = 0.553, *ƞp* ^2^ = 0.002; the main effect of Condition was not significant, *F*(1, 223) = 2.844, *p* = 0.093, *ƞp* ^2^ = 0.013; the main effect of Electrode was significant, *F*(1.646, 223) = 9.619, *p* < 0.001, *ƞp* ^2^ = 0.041; the Group × Condition interaction was not significant, *F*(1, 223) = 0.156, *p* = 0.693, *ƞp* ^2^ = 0.001; the Group × Electrode interaction was not significant, *F*(1.646, 223) =  0.423, *p* =0.616, *ƞp* ^2^ =  0.002; the Condition × Electrode interaction was not significant, *F*(1.892, 223) = 0.443, *p* = 0.632, *ƞp* ^2^ = 0.002; the Group × Condition × Electrode interaction was significant, *F*(1.892, 223) = 3.349, *p* = 0.039, *ƞp* ^2^ = 0.015. Simple effects analysis revealed that in the Healthy Control Group, the N2 latency induced by the congruent condition at the Fz electrode was significantly longer than that induced by the incongruent condition. In the Healthy Control Group, under the incongruent condition, the N2 latency induced at the Pz and Cz electrodes was significantly longer than at the Fz electrode, Fz < Cz < Pz. In the Depressive Symptom Group, under the congruent condition, the N2 latency induced at the Pz and Cz electrodes was significantly longer than at the Fz electrode, Fz < Cz < Pz. These results suggest that there is no significant difference in N2 latency between the Depressive Symptom Group and the Healthy Control Group in the Stroop task.

##### 3.2.2.4 Interference Suppression of the P3 Component

In the Stroop task, a 2 (Group) × 2 (Condition) × 3 (Electrode) repeated measures ANOVA was conducted with P3 amplitude as the dependent variable. The results (Table 3, Figure 3) showed that the main effect of Group was not significant, *F*(1, 223) = 2.302, *p* = 0.131, *ƞp* ^2^ = 0.010; the main effect of Condition was not significant, *F*(1, 223) = 3.140, *p* = 0.078, *ƞp* ^2^ = 0.014; the main effect of Electrode was significant, *F*(1.931, 223) = 78.421, *p* < 0.001, *ƞp* ^2^ = 0.260; the Group × Condition interaction was significant, *F*(1, 223) = 5.829, *p* = 0.017, *ƞp* ^2^ = 0.025; the Group × Electrode interaction was not significant, *F*(1.931, 223) = 2.182, *p* = 0.116, *ƞp* ^2^ = 0.010; the Condition × Electrode interaction was not significant, *F*(1.930, 223) = 0.080, *p* = 0.917, *ƞp* ^2^ < 0.001; the Group × Condition × Electrode interaction was significant, *F*(1.930, 223) = 3.700, *p* = 0.027, *ƞp* ^2^ = 0.016. Simple effects analysis showed that in the Healthy Control Group, the P3 amplitude induced at the Fz, Cz, and Pz electrodes under the incongruent condition was significantly larger than in the Depressive Symptom Group. In the Healthy Control Group, the P3 amplitude induced at the Cz and Pz electrodes under the incongruent condition was significantly larger than that induced under the congruent condition. The Healthy Control Group showed significant differences across all electrodes under both congruent and incongruent conditions, with Pz < Cz < Fz. In the Depressive Symptom Group, under the congruent condition, the P3 amplitude at the Fz electrode was significantly larger than at the Cz and Pz electrodes, with Fz < Cz < Pz under the incongruent condition. These results suggest that in the Stroop incongruent condition, the P3 amplitude at the Fz, Cz, and Pz electrodes in the Healthy Control Group was larger than in the Depressive Symptom Group.

In the Stroop task, a 2 (Group) × 2 (Condition) × 3 (Electrode) repeated measures ANOVA was conducted with P3 latency as the dependent variable. The results (Table 3, Figure 3) showed that the main effect of Group was not significant, *F*(1, 223) = 1.507, *p* = 0.221, *ƞp* ^2^ = 0.007; the main effect of Condition was not significant, *F*(1, 223) = 0.991, *p* = 0.321, *ƞp* ^2^ = 0.004; the main effect of Electrode was significant, *F*(1.733, 223) = 16.286, *p* < 0.001, *ƞp* ^2^ = 0.068; the Group × Condition interaction was not significant, *F*(1, 223) = 0.048, *p* = 0.826, *ƞp* ^2^ < 0.001; the Group × Electrode interaction was not significant, *F*(1.733, 223) = 2.929, *p* = 0.062, *ƞp* ^2^ = 0.013; the Condition × Electrode interaction was not significant, *F*(1.854, 223) = 0.629, *p* = 0.522, *ƞp* ^2^ = 0.003; the Group × Condition × Electrode interaction was not significant, *F*(1.854, 223) = 0.969, *p* = 0.375, *ƞp* ^2^ = 0.004. These results suggest that there is no significant difference in P3 latency between the Depressive Symptom Group and the Healthy Control Group in the Stroop task.

#### 3.2.3 Relationship Between Inhibitory Function Difference Indices in Depressive Symptom Students and Healthy Control Students and Depressive Symptoms

The inhibitory function differential indices between depressive symptom students and healthy control students were Nogo accuracy, Stroop incongruent accuracy, Stroop congruent response time, Stroop incongruent response time, N2 amplitude (Fz) under Go and Nogo conditions, and P3 amplitude (Fz, Cz, Pz) under the Stroop incongruent condition. Depressive symptom scores were significantly negatively correlated with Nogo accuracy (*r* = −0.192, *p* = 0.004), Stroop incongruent accuracy (*r* = −0.176, *p* = 0.008), P3 amplitude of Fz potentials under the Stroop incongruent condition (*r* = −0.183, *p* = 0.006), P3 amplitude of Cz potentials under the Stroop incongruent condition (*r* = −0.191, *p* = 0.004), and P3 amplitude of Pz potentials under the Stroop incongruent condition (*r* = −0.188, *p* = 0.005). Depressive symptom scores were significantly positively correlated with Stroop congruent response time (*r* = 0.243, *p* < 0.001), Stroop incongruent response time (*r* = 0.298, *p* < 0.001), N2 amplitude of Fz potentials under the Go condition (*r* = 0.226, *p* = 0.001), and N2 amplitude of Fz potentials under the Nogo condition (*r* = 0.228, *p* = 0.001).

To further investigate the explanatory power of the inhibitory function differential indices on depressive symptoms, multiple linear regression analysis (stepwise method) was conducted with depressive symptom scores as the dependent variable and inhibitory function differential indices as independent variables. The results showed (Table 4) that the regression model passed the significance test (*F* = 11.787, *p* < 0.001, *R* ^2^ = 0.176). Stroop incongruent response time, Nogo accuracy, P3 amplitude of Fz potentials under the Stroop incongruent condition, and N2 amplitude of Fz potentials under the Nogo condition were found to predict depressive symptom scores, while other indices were excluded. The tolerance for each independent variable was > 0.1, and the VIF values were all < 5, indicating that multicollinearity is unlikely to have influenced the results. The results suggest that Stroop incongruent response time, Nogo accuracy, P3 amplitude of Fz potentials under the Stroop incongruent condition, and N2 amplitude of Fz potentials under the Nogo condition are specific inhibitory function indices for depressive symptoms and may serve as key observational indicators for the early identification of depressive symptoms in college students.

**TABLE 4:** Multiple linear regression analysis of the inhibitory function differentiation indices explaining depressive symptoms in depressive symptom and healthy control students (*n* = 225).

Independent variable	*B*	95% CI		Beta	Coefficient significance test		SE	Collinearity diagnostics	
		Lower	Upper		*t*‐value	*t*‐value		Tolerance	VIF
Stroop incongruent Response time	22.036	11.928	32.144	0.267	4.296	< 0.001	5.129	0.968	1.033
Nogo accuracy	−0.123	−0.201	−0.045	−0.191	−3.101	0.002	0.040	0.990	1.010
P3 amplitude of Fz potentials under the Stroop incongruent condition	−0.438	−0.793	−0.083	−0.151	−2.434	0.016	0.180	0.972	1.029
N2 amplitude of Fz potentials under the Nogo condition	0.212	0.028	0.395	0.143	2.272	0.024	0.093	0.941	1.063

*Note:* Model summary *F* = 11.787 (*p* < 0.001), *R* = 0.420, *R* ^2^ = 0.176, adjusted *R* ^2^ = 0.162.

Abbreviations: *B*, unstandardized coefficients; Beta, standardized coefficients; CI, confidence interval; SE, standard error.

### 3.3 The Relationship Between Physical Exercise and Depressive Symptoms

#### 3.3.1 The Relationship Between Physical Exercise Volume and Depressive Symptom Scores and Inhibition Function‐Specific Indicators

**FIGURE 4:**
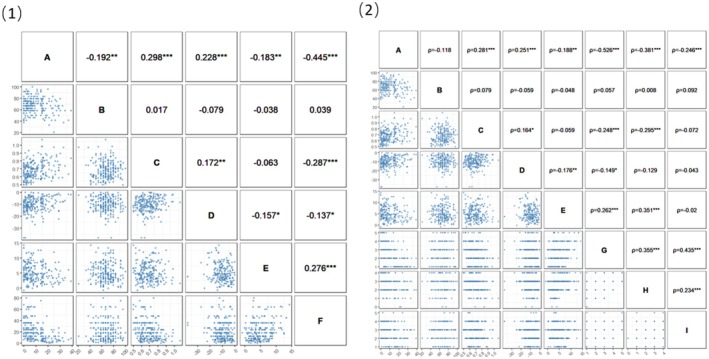
Relationships between physical exercise volume, intensity, duration, frequency, depressive symptom scores, and inhibitory function‐specific indicators (*n* = 225). *p < 0.05, **p < 0.01, ***p < 0.001. A represents depressive symptom scores; B represents Nogo accuracy; C represents Stroop incongruent reaction time; D represents the N2 amplitude of Fz potentials under the Nogo condition; E represents the P3 amplitude of Fz potentials under the Stroop incongruent condition; F represents physical exercise volume; G represents intensity; H represents duration; I represents frequency.

As shown in Figure 4 (1), physical exercise volume is significantly negatively correlated with depressive symptom scores (*r* = −0.445, *p* < 0.001). The specific inhibitory function indices for depressed students are Nogo accuracy, Stroop incongruent reaction time, N2 amplitude of Fz potentials under the Nogo condition, and P3 amplitude of Fz potentials under the Stroop incongruent condition. Physical exercise volume is positively correlated with Nogo accuracy (*r* = 0.039, *p* = 0.559), but this correlation is not statistically significant. Physical exercise volume is significantly negatively correlated with Stroop incongruent reaction time (*r* = −0.287, *p* < 0.001) and N2 amplitude of Fz potentials under the Nogo condition (*r* = −0.137, *p* = 0.041). Physical exercise volume is significantly positively correlated with P3 amplitude of Fz potentials under the Stroop incongruent condition (*r* = 0.276, *p* < 0.001). The results suggest that with an increase in physical exercise volume, Stroop incongruent reaction time shortens, N2 amplitude of Fz potentials under the Nogo condition increases, P3 amplitude of Fz potentials under the Stroop incongruent condition increases, and depressive symptom scores decrease.

#### 3.3.2 Relationship Between Different Components of Physical Exercise and Depressive Symptom Scores, and Inhibitory Function‐Specific Indicators

As shown in Figure 4 (2), intensity is significantly negatively correlated with depressive symptom scores (*ρ* = −0.526, *p* < 0.001). Intensity is positively correlated with Nogo accuracy (*ρ* = 0.057, *p* = 0.354), but this correlation is not statistically significant. Intensity is significantly negatively correlated with Stroop incongruent reaction time (*ρ* = −0.248, *p* < 0.001) and N2 amplitude of Fz potentials under the Nogo condition (*ρ* = −0.149, *p* = 0.025). Intensity is significantly positively correlated with P3 amplitude of Fz potentials under the Stroop incongruent condition (*ρ* = 0.262, *p* < 0.001). These results suggest that with increasing exercise intensity, Stroop incongruent reaction time decreases, N2 amplitude of Fz potentials under the Nogo condition increases, P3 amplitude of Fz potentials under the Stroop incongruent condition increases, and depressive symptom scores decrease.

As shown in Figure 4 (2), duration per session is significantly negatively correlated with depressive symptom scores (*ρ* = −0.381, *p* < 0.001). Duration is positively correlated with Nogo accuracy (*ρ* = 0.008, *p* = 0.902), and negatively correlated with N2 amplitude of Fz potentials under the Nogo condition (*ρ* = −0.129, *p* = 0.078), but neither correlation is statistically significant. Duration is significantly negatively correlated with Stroop incongruent reaction time (*ρ* = −0.294, *p* < 0.001). Duration is significantly positively correlated with P3 amplitude of Fz potentials under the Stroop incongruent condition (*ρ* = 0.351, *p* < 0.001). These results suggest that with increasing exercise duration per session, Stroop incongruent reaction time decreases, P3 amplitude of Fz potentials under the Stroop incongruent condition increases, and depressive symptom scores decrease.

As shown in Figure 4 (2), frequency is significantly negatively correlated with depressive symptom scores (*ρ* = −0.246, *p* < 0.001). Frequency does not show significant correlations with the inhibitory function‐specific indicators (*p* > 0.05 for all). These results suggest that with increasing exercise frequency, depressive symptom scores decrease.

#### 3.3.3 Differences in Depressive Symptom Scores and Inhibitory Function‐Specific Indicators Across Different Levels of Physical Exercise Components

##### 3.3.3.1 Differences in Depressive Symptom Scores and Inhibitory Function‐Specific Indicators Among College Students Engaging in Different Intensity Levels of Physical Exercise

According to the scale items, physical exercise intensity can be classified into mild exercise (Level 1), low‐intensity exercise (Level 2), moderate‐intensity sustained exercise (Level 3), high‐intensity non‐sustained exercise (Level 4), and high‐intensity sustained exercise (Level 5). Depressive symptoms, Stroop incongruent reaction time, N2 amplitude of Fz potentials under the Nogo condition, and P3 amplitude of Fz potentials under the Stroop incongruent condition were significantly correlated with exercise intensity. The differences across various intensity levels were further explored.

Intensity is an influencing factor for depressive symptoms (Tables 5 and 6), with significant differences found between moderate‐intensity sustained exercise, high‐intensity non‐sustained exercise, high‐intensity sustained exercise, and mild exercise and low‐intensity exercise (*p* < 0.05), with larger effects. Intensity is an influencing factor for Stroop incongruent reaction time, with significant differences observed between moderate‐intensity sustained exercise, high‐intensity non‐sustained exercise, high‐intensity sustained exercise, and low‐intensity exercise (*p* < 0.05), with larger effects. Intensity is not an influencing factor for N2 amplitude of Fz potentials under the Nogo condition. Intensity is an influencing factor for P3 amplitude of Fz potentials under the Stroop incongruent condition, with significant differences between high‐intensity non‐sustained exercise and mild exercise and low‐intensity exercise (*p* < 0.05), with larger effects. The results suggest that intensity is an influencing factor for depressive symptoms and interference inhibition. Exercise of moderate intensity or higher is recommended for depressive symptoms and interference inhibition, while high‐intensity non‐sustained exercise provides greater benefits for cognitive neural processing related to interference inhibition.

**TABLE 5:** Differences in depressive symptoms and inhibition‐specific indices across different physical exercise components (*n* = 225).

Variables	Depressive symptom scores	Stroop incongruent reaction time	N2 amplitude of Fz potentials under the Nogo condition	P3 amplitude of Fz potentials under the Stroop incongruent condition
Intensity				
Level 1 (*n* = 39)	7.564 ± 9.433	0.706 ± 0.103	−8.654 ± 5.314	3.892 ± 2.025
Level 2 (*n* = 62)	13.242 ± 9.186	0.732 ± 0.117	−9.073 ± 5.244	4.528 ± 2.666
Level 3 (*n* = 72)	6.333 ± 5.814	0.679 ± 0.093	−11.270 ± 6.049	5.239 ± 2.955
Level 4 (*n* = 40)	5.475 ± 5.354	0.653 ± 0.092	−10.995 ± 7.278	6.613 ± 3.493
Level 5 (*n* = 12)	2.917 ± 3.965	0.611 ± 0.105	−10.511 ± 5.445	6.642 ± 4.209
*F*‐value, *p*‐value	*F* = 23.706, *p* < 0.001	*F* = 6.168, *p* < 0.001	*F* = 1.986, *p* = 0.098	*F* = 5.809, *p* < 0.001
Duration				
Level 1 (*n* = 2)	21.500 ± 0.707	0.766 ± 0.104	—	2.986 ± 1.457
Level 2 (*n* = 17)	14.588 ± 8.456	0.761 ± 0.133	—	2.979 ± 1.007
Level 3 (*n* = 66)	13.424 ± 9.662	0.719 ± 0.106	—	4.275 ± 2.838
Level 4 (*n* = 117)	7.821 ± 7.786	0.672 ± 0.099	—	5.661 ± 3.122
Level 5 (*n* = 23)	5.391 ± 6.058	0.641 ± 0.084	—	6.650 ± 2.891
*F*‐value, *p*‐value	*F* = 8.899, *p* < 0.001	*F* = 5.811, *p* < 0.001	—	*F* = 6.587, *p* < 0.001
Frequency				
Level 1 (*n* = 27)	14.778 ± 7.470	—	—	—
Level 2 (*n* = 67)	10.791 ± 9.207	—	—	—
Level 3 (*n* = 82)	9.523 ± 1.052	—	—	—
Level 4 (*n* = 46)	6.018 ± 0.887	—	—	—
Level 5 (*n* = 3)	13.333 ± 11.015	—	—	—
*F*‐value, *p*‐value	*F* = 4.138, *p* = 0.003	—	—	—

*Note:* Physical exercise intensity can be classified into mild exercise (Level 1), low‐intensity exercise (Level 2), moderate‐intensity sustained exercise (Level 3), high‐intensity non‐sustained exercise (Level 4), and high‐intensity sustained exercise (Level 5). Exercise duration can be classified into < 10 min (Level 1), 11–20 min (Level 2), 21–30 min (Level 3), 31–59 min (Level 4), and more than 60 min (Level 5). Exercise frequency can be classified into < 1 time/month (Level 1), 2–3 times/month (Level 2), 1–2 times/week (Level 3), 3–5 times/week (Level 4), and approximately 7 times/week (Level 5).

**TABLE 6:** Post hoc multiple comparisons of depressive symptom scores and inhibition function specific indicators across different levels of physical exercise components (*n* = 225).

Variables	Group comparison	Cohen's *d* (95% CI lower, upper)		
		Depressive symptom scores	Stroop incongruent reaction time	P3 amplitude of Fz potentials under the Stroop incongruent condition
Intensity	Level 1 vs. Level 2	0.580 (−0.005, 1.165)	−0.261 (−0.841, 0.320)	−0.217 (−0.798, 0.363)
	Level 1 vs. Level 3	1.507 (0.907, 2.106)	0.260 (−0.304, 0.825)	−0.461 (−1.028, 0.106)
	Level 1 vs. Level 4	1.622 (0.947, 2.297)	0.515 (−0.127, 1.157)	−0.931 (−1.581, −0.280)
	Level 1 vs. Level 5	1.965 (0.992, 2.938)	0.921 (−0.023, 1.865)	−0.941 (−1.885, 0.004)
	Level 2 vs. Level 3	0.927 (0.420, 1.434)	0.521 (0.025, 1.017)	−0.243 (−0.736, 0.249)
	Level 2 vs. Level 4	1.042 (0.450, 1.634)	0.775 (0.191, 1.360)	−0.714 (−1.297, −0.130)
	Level 2 vs. Level 5	1.385 (0.472, 2.299)	1.182 (0.273, 2.090)	−0.723 (−1.623, 0.176)
	Level 3 vs. Level 4	0.115 (−0.444, 0.675)	0.254 (−0.306, 0.815)	−0.470 (−1.033, 0.093)
	Level 3 vs. Level 5	0.458 (−0.428, 1.345)	0.661 (−0.228, 1.549)	−0.480 (−1.366, 0.407)
	Level 4 vs. Level 5	0.343 (−0.591, 1.278)	0.406 (−0.529, 1.341)	−0.010 (−0.943, 0.924)
Duration	Level 1 vs. Level 2	0.836 (−1.287, 2.958)	0.052 (−2.068, 2.172)	0.002 (−2.117, 2.122)
	Level 1 vs. Level 3	0.976 (−1.063, 3.016)	0.460 (−1.576, 2.496)	−0.444 (−2.480, 1.592)
	Level 1 vs. Level 4	1.654 (−0.381, 3.688)	0.918 (−1.108, 2.944)	−0.921 (−2.947, 1.105)
	Level 1 vs. Level 5	1.948 (−0.159, 4.054)	1.217 (−0.880, 3.314)	−1.262 (−3.359, 0.836)
	Level 2 vs. Level 3	0.141 (−0.631, 0.912)	0.408 (−0.365, 1.181)	−0.446 (−1.220, 0.327)
	Level 2 vs. Level 4	0.818 (0.074, 1.562)	0.866 (0.121, 1.611)	−0.923 (−1.670, −0.177)
	Level 2 vs. Level 5	1.112 (0.193, 2.031)	1.165 (0.244, 2.085)	−1.264 (−2.187, −0.341)
	Level 3 vs. Level 4	0.677 (0.231, 1.124)	0.458 (0.017, 0.899)	−0.477 (−0.918, −0.036)
	Level 3 vs. Level 5	0.971 (0.272, 1.670)	0.757 (0.063, 1.451)	−0.818 (−1.513, −0.123)
	Level 4 vs. Level 5	0.294 (−0.354, 0.942)	0.299 (−0.349, 0.947)	−0.341 (−0.989, 0.308)
Frequency	Level 1 vs. Level 2	0.464 (−0.186, 1.113)	—	—
	Level 1 vs. Level 3	0.671 (0.035, 1.306)	—	—
	Level 1 vs. Level 4	0.922 (0.224, 1.621)	—	—
	Level 1 vs. Level 5	0.168 (−1.558, 1.894)	—	—
	Level 2 vs. Level 3	0.207 (−0.261, 0.675)	—	—
	Level 2 vs. Level 4	0.459 (−0.088, 1.005)	—	—
	Level 2 vs. Level 5	−0.296 (−1.970, 1.378)	—	—
	Level 3 vs. Level 4	0.252 (−0.272, 0.775)	—	—
	Level 3 vs. Level 5	−0.503 (−2.171, 1.166)	—	—
	Level 4 vs. Level 5	−0.754 (−2.447, 0.938)	—	—

*Note:* Cohen's *d* represents the standardized effect size; CI stands for confidence interval; lower indicates the lower limit; upper indicates the upper limit. Intensity is categorized as mild exercise (Level 1), low‐intensity exercise (Level 2), moderate‐intensity sustained exercise (Level 3), high‐intensity non‐sustained exercise (Level 4), and high‐intensity sustained exercise (Level 5); Duration is categorized as < 10 min (Level 1), 11–20 min (Level 2), 21–30 min (Level 3), 31–59 min (Level 4), and > 60 min (Level 5); Frequency is categorized as < 1 time/month (Level 1), 2–3 times/month (Level 2), 1–2 times/week (Level 3), 3–5 times/week (Level 4), and about 7 times/week (Level 5).

##### 3.3.3.2 Differences in Depressive Symptom Scores and Inhibition Function‐Specific Indicators Among College Students Engaging in Different Durations of Physical Exercise

Based on the questionnaire items, the duration of each physical exercise session is categorized as < 10 min (level 1), 11–20 min (level 2), 21–30 min (level 3), 31–59 min (level 4), and more than 60 min (level 5). Depressive symptoms, Stroop incongruent reaction time, and P3 amplitude of Fz potentials under the Stroop incongruent condition are significantly correlated with duration, exploring the differences at different duration levels.

Duration is a significant factor for depressive symptoms (Tables 5 and 6). There are significant differences between 21–30 min, 31–59 min, and more than 60 min compared with 11–20 min (*p* < 0.05), with larger effects; significant differences also exist between 31–59 min, more than 60 min, and 21–30 min (*p* < 0.05), with larger effects. Duration is a significant factor for Stroop incongruent reaction time. There are significant differences between 31–59 min, more than 60 min, and 11–20 min and 21–30 min (*p* < 0.05), with larger effects. Duration is a significant factor for the P3 amplitude of Fz potentials under the Stroop incongruent condition. Significant differences exist between 31–59 min, more than 60 min, and 11–20 min and 21–30 min (*p* < 0.05), with larger effects. The results suggest that duration is an influencing factor for depressive symptoms and interference inhibition. For depressive symptoms and interference inhibition, exercise lasting more than 30 min is recommended.

##### 3.3.3.3 Differences in Depressive Symptom Scores Among College Students Engaging in Different Frequencies of Physical Exercise

Based on the questionnaire items, the frequency of physical exercise is categorized as < 1 time/month (level 1), 2–3 times/month (level 2), 1–2 times/week (level 3), 3–5 times/week (level 4), and approximately 7 times/week (level 5). Depressive symptoms are significantly correlated with frequency, exploring the differences at different frequency levels.

Frequency is an influencing factor for depressive symptoms (Tables 5 and 6). There are significant differences between 1–2 times/week, 3–5 times/week, and 1 time/month (*p* < 0.05), with larger effects. The results suggest that frequency is an influencing factor for depressive symptoms. Exercise 1–2 times/week and 3–5 times/week are recommended for depressive symptoms, with the optimal frequency being 3–5 times/week.

## 4 Discussion

The results of this study indicate that college students with depressive symptoms exhibit impaired inhibitory control, including deficits in both response inhibition and interference inhibition. In terms of response inhibition, these students showed a decreased Nogo correct response rate during behavioral tasks, reflecting their difficulty in inhibiting responses when faced with situations that require response suppression. In terms of cognitive processing mechanisms, the N2 amplitude of Fz potentials under the Nogo condition was reduced, suggesting a decline in conflict monitoring and inhibitory control in individuals with depression. With regard to interference inhibition, depressive symptom students displayed prolonged Stroop incongruent reaction times, indicating difficulties in filtering out irrelevant information. These individuals require more time to respond automatically to interference, and they are less efficient at mobilizing cognitive resources to suppress irrelevant responses [33, 34]. In terms of cognitive neural processing, the reduced P3 amplitude further reflects impairments in the brain's ability to mobilize cognitive resources for inhibition and conflict resolution when confronted with interference, leading to a diminished capacity for effective cognitive resource allocation [35]. Previous research also supports these findings. Compared to healthy individuals, those with depression perform more poorly on tasks of response and interference inhibition [36, 37]. They exhibit early deficits in conflict monitoring [13], early stage information processing [38], late‐stage conflict resolution, and behavioral inhibition [14]. A possible explanation for these impairments is that inhibitory control is regulated by the frontal brain regions. The N2 component is primarily localized in the anterior regions around the frontal‐central area and originates from the anterior cingulate cortex, while the P3 component is mainly distributed in the cortical areas of the parietal and frontal‐central regions. The anatomical origins of these components involve brain structures such as the frontal lobe, cingulate gyrus, hippocampus, and the temporo‐parietal junction. Depressive individuals exhibit structural and functional abnormalities in multiple brain regions, including reduced volumes of the anterior cingulate gyrus, orbitofrontal cortex, dorsolateral prefrontal cortex, ventromedial prefrontal cortex, and caudate nucleus [39, 40]. Additionally, damage to the frontal white matter fiber tracts [41] further impairs inhibitory control, leading to task performance declines and abnormal cognitive processing.

This study found that as the physical exercise volume increased, the Depressive Symptom Scores decreased. The intensity, duration, and frequency of physical exercise were identified as significant factors influencing depressive symptoms. The recommended exercise intensity is moderate or higher, with a recommended duration of more than 30 min per session and a frequency of 1–2 times per week or 3–5 times per week, with the optimal frequency being 3–5 times per week. Previous studies also support this finding, indicating a negative correlation between physical exercise and depressive symptoms. Regular physical exercise can prevent and alleviate depressive symptoms and is closely related to exercise modality, intensity, frequency, and duration [42, 43, 44]. The World Health Organization recommends that adults aged 18–64 engage in at least 150 min of moderate‐intensity aerobic exercise per week, or at least 75 min of vigorous‐intensity aerobic exercise, with each session lasting at least 10 min [45]. A 13‐year cohort study involving 43,863 Swedish adults found that participants who engaged in physical exercise for ≥ 300 min per week had a 29% lower risk of depression [46]. Cross‐sectional studies have found a significant negative correlation between moderate to vigorous physical activity and the severity of depressive symptoms, with recommendations for individuals with depressive symptoms to engage in moderate to vigorous physical activity for at least 30 min per day [47]. There is a U‐shaped association between exercise frequency, duration, and mental health, with the greatest improvement in mental health observed with 10–15 sessions per month, each lasting 40–50 min [48]. Systematic reviews have also found that exercise three times per week provides significant benefits for depressive symptoms [49]. Therefore, the exercise dosage must consider the combination of different exercise components, including modality, intensity, duration, frequency, and periodization, which collectively determine the overall effectiveness of an exercise program. For example, high‐intensity exercise may require shorter durations and lower frequencies, while moderate‐intensity exercise may require longer durations and higher frequencies to achieve the same health benefits. Although the association between physical exercise and depression is strong, the mechanisms underlying the antidepressant effects of physical exercise remain unclear. Potential mechanisms include: activation of the endocannabinoid system to stimulate the release of endorphins, improving mood and alleviating pain [50]; promotion of the release of mood‐regulating neurotransmitters such as dopamine, serotonin, and norepinephrine in the brain [51, 52], leading to positive emotions; increased cerebral blood flow, restructuring of brain structure, and enhancement of the volume and white matter integrity in the frontal–parietal regions [43]; activation of the prefrontal cortex, upregulation of brain‐derived neurotrophic factor expression, promoting neuronal growth and survival, and synaptic formation and repair [23, 53], enhancing cognitive function and improving emotional regulation.

This study found that as the physical exercise volume increased, interference suppression, as measured by the Stroop incongruent reaction time, shortened, and the P3 amplitude of the Fz potential under the Stroop incongruent condition increased, while Depressive Symptom Scores decreased. Physical exercise intensity and duration were identified as significant factors influencing interference suppression. The recommended intensity is moderate or higher, with the cognitive‐neuroprocessing benefits of high‐intensity non‐sustained exercise being particularly beneficial, and the recommended duration is more than 30 min. Previous research supports this result, showing a positive correlation between physical activity and inhibitory function [8, 54, 55]. Exercise specifically improves executive function and brain activation, with a dose–response relationship between exercise and changes in inhibitory function [56]. Studies have found that only moderate‐intensity exercise leads to significant cognitive improvements [57]. Moderate‐intensity exercise can effectively release catecholamines, increase biological arousal in the central nervous system, and enhance the efficiency of cognitive resource allocation [58, 59], thereby improving inhibitory function and increasing positive emotions. High‐intensity exercise leads to a greater increase in brain‐derived neurotrophic factor (BDNF), which is more beneficial in exerting the neural structural and functional regulatory effects of exercise [60]. This promotes neurogenesis and synaptic plasticity, improving brain structure and functional abnormalities [43], thereby enhancing inhibitory function and emotional regulation. These pathways ultimately improve symptoms of mood depression, diminished willpower, and cognitive impairment in individuals with depression. In terms of exercise duration, a study found that after a 3‐month aerobic exercise intervention for children, the cognitive scores in the high‐dose group (40 min per day) improved significantly, while the cognitive scores in the low‐dose group (20 min per day) did not differ significantly from the control group [61]. A meta‐analysis of 19 studies involving 5038 participants confirmed that long‐term exercise promotes multiple aspects of executive function in children and adolescents, especially inhibitory control. Interventions lasting < 90 min improved executive function, whereas those lasting 90 min or more showed no significant improvements [54]. A meta‐analysis on depression patients also indicated that aerobic exercise significantly improves overall cognitive function and executive function, recommending moderate to vigorous (mixed) intensity, three times per week, with each session lasting < 45 min, and the intervention duration not exceeding 12 weeks. Therefore, for individuals with depressive symptoms who exhibit impaired interference suppression, moderate or higher exercise intensity can improve task performance, while high‐intensity non‐sustained exercise can enhance the effective mobilization and allocation of cognitive resources, as well as inhibitory and conflict resolution abilities. Exercise intervention plans should be designed to match the duration based on different exercise intensity levels. Moreover, this study found that as Physical Exercise Volume and intensity increased, the N2 amplitude of Fz potentials under the Nogo condition, which reflects response inhibition, increased. However, no significant differences were observed between different intensity levels. The increase in Physical Exercise Volume and intensity may enhance individuals' conflict monitoring and inhibitory abilities. The lack of significant differences between different intensity levels may be due to the fact that the Go/Nogo task used in this study, which reflects response inhibition, is relatively easy, potentially leading to a “ceiling effect,” with small individual differences in task performance. This limits the variability of the results, making it difficult to detect differences between different intensity levels. Future research could increase task difficulty by adjusting the complexity of stimuli or the response requirements, thereby increasing individual performance variability to more accurately observe the effects of different levels of Physical Exercise Volume and intensity.

### 4.1 Limitations

This study is designed as an observational study, and longitudinal research is needed in the future to further validate the findings. The participants included in this study were college students with depressive symptoms, but they were not clinically diagnosed, which limits the generalizability of the results. This study primarily focused on electrophysiological activity and did not explore the morphological changes in brain regions involved in inhibitory function. Future research could combine magnetic resonance imaging (MRI) technology to further investigate these changes. Additionally, this study only examined the effects of different levels of exercise intensity, frequency, and duration on depressive symptoms and specific inhibitory function indicators, without considering exercise form and cycle, which may introduce some bias. Future studies should consider a comprehensive set of exercise elements and combinations to provide more precise exercise intervention plans.

## 5 Conclusion

College students with depressive symptoms exhibit impaired inhibitory functions, including decreased performance in response inhibition and interference inhibition tasks, along with diminished cognitive processing abilities. These impairments could serve as key indicators for early detection of depressive symptoms in university students. Physical exercise volume is closely associated with depressive symptoms, and exercise intensity, duration, and frequency are significant influencing factors. It is recommended that exercise intensity be at least moderate, with a duration of 30 min or more, and a frequency of 1–2 times per week or 3–5 times per week, with the optimal frequency being 3–5 times per week. Physical exercise volume is also closely linked to response inhibition and interference inhibition, with exercise intensity and duration being key factors influencing interference inhibition. It is recommended that the exercise intensity be at least moderate, with high‐intensity non‐sustained exercise showing better benefits in cognitive processing. The recommended duration is also 30 min or more. When designing exercise programs, it is essential to consider the combination of various exercise components, and based on individual differences in depressive symptoms, personalized and targeted exercise interventions should be developed.

## Author Contributions

Shufan Li: conceptualization, methodology, writing – original draft, data curation. Shuqi Jia: software, investigation. Somang Yun: data curation, investigation. Zhaohui Guo: investigation. Xing Wang: investigation. Qingwen Zhang: writing – reviewing and editing.

## Ethics Statement

For experiments involving human participants, informed consent has been obtained from all participants (all adults) in this study. Our study was approved by the ethical committee of Shanghai University of Sport (102772023RT075). All methods were carried out in accordance with relevant guidelines and regulations.

## Conflicts of Interest

The authors declare no conflicts of interest.

## Supporting information


Data S1.

## Data Availability

The datasets used and/or analyzed during the current study are available from the corresponding author on reasonable request.
