# Supplementary material for: The Relationship Between Different Components and Levels of Physical Exercise, Depressive Symptoms, Inhibitory Control, and Possible Cognitive Neural Mechanisms in College Students
Source: CNS Neurosci Ther. 2025 Jul 31;31(8):e70520. doi: 10.1111/cns.70520 (PMC12313544; doi:10.1111/cns.70520)
Supplement: Supplementary file 1 — Data S1. [file CNS-31-e70520-s001.pdf]

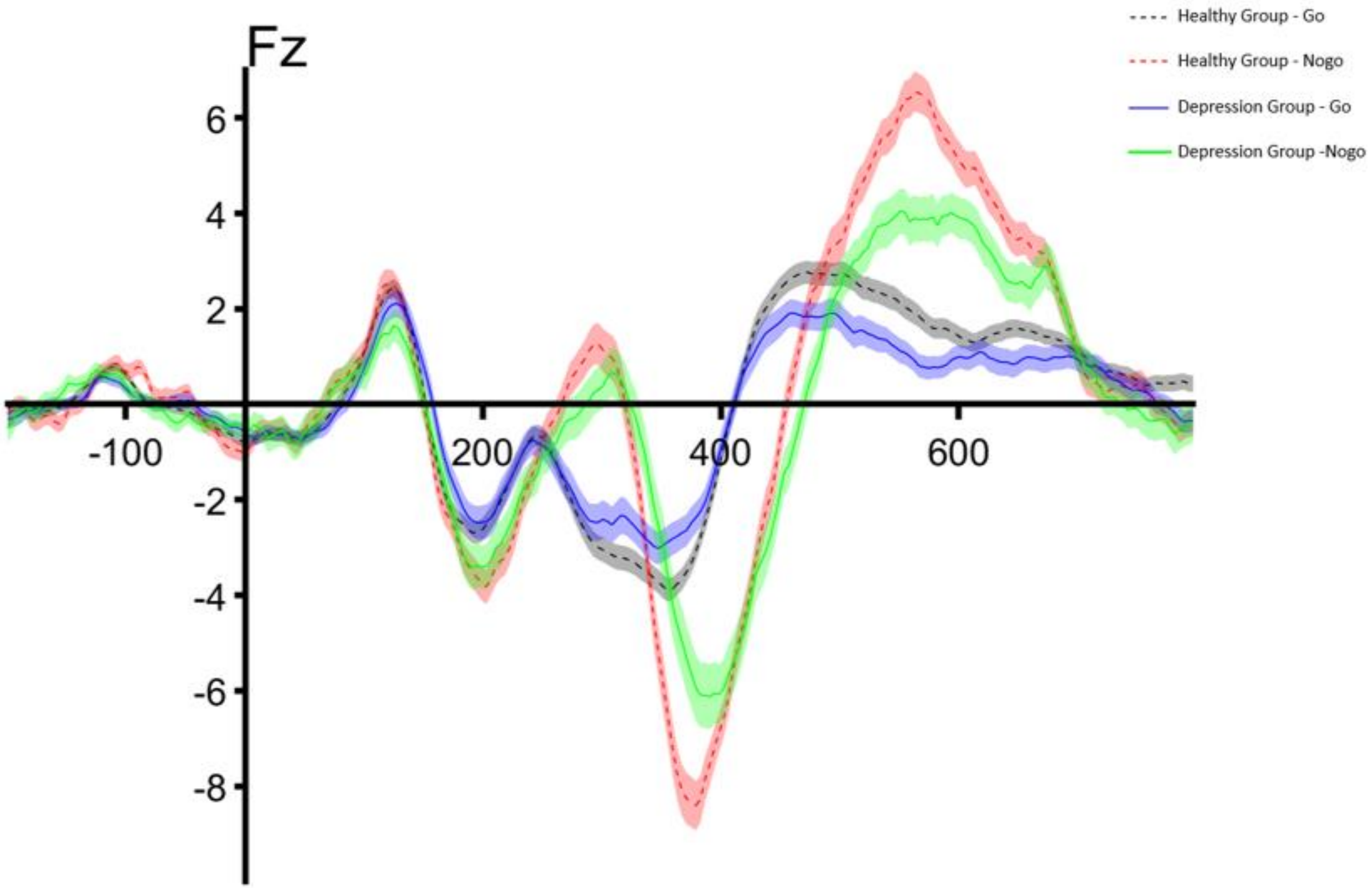

Figure 3 Fz potentials induced by the Go/No-Go task

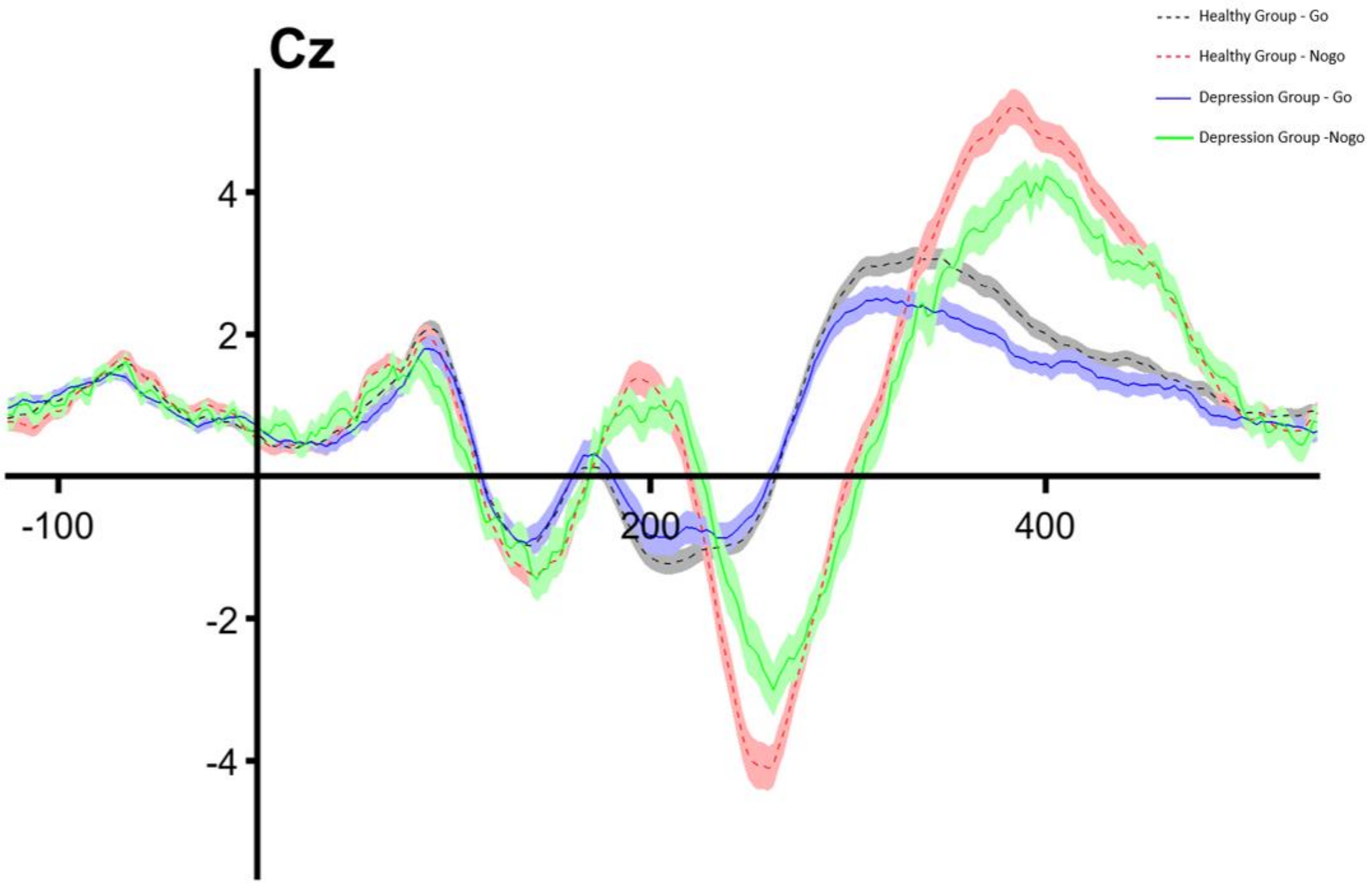

Figure 3 Cz potentials induced by the Go/No-Go task

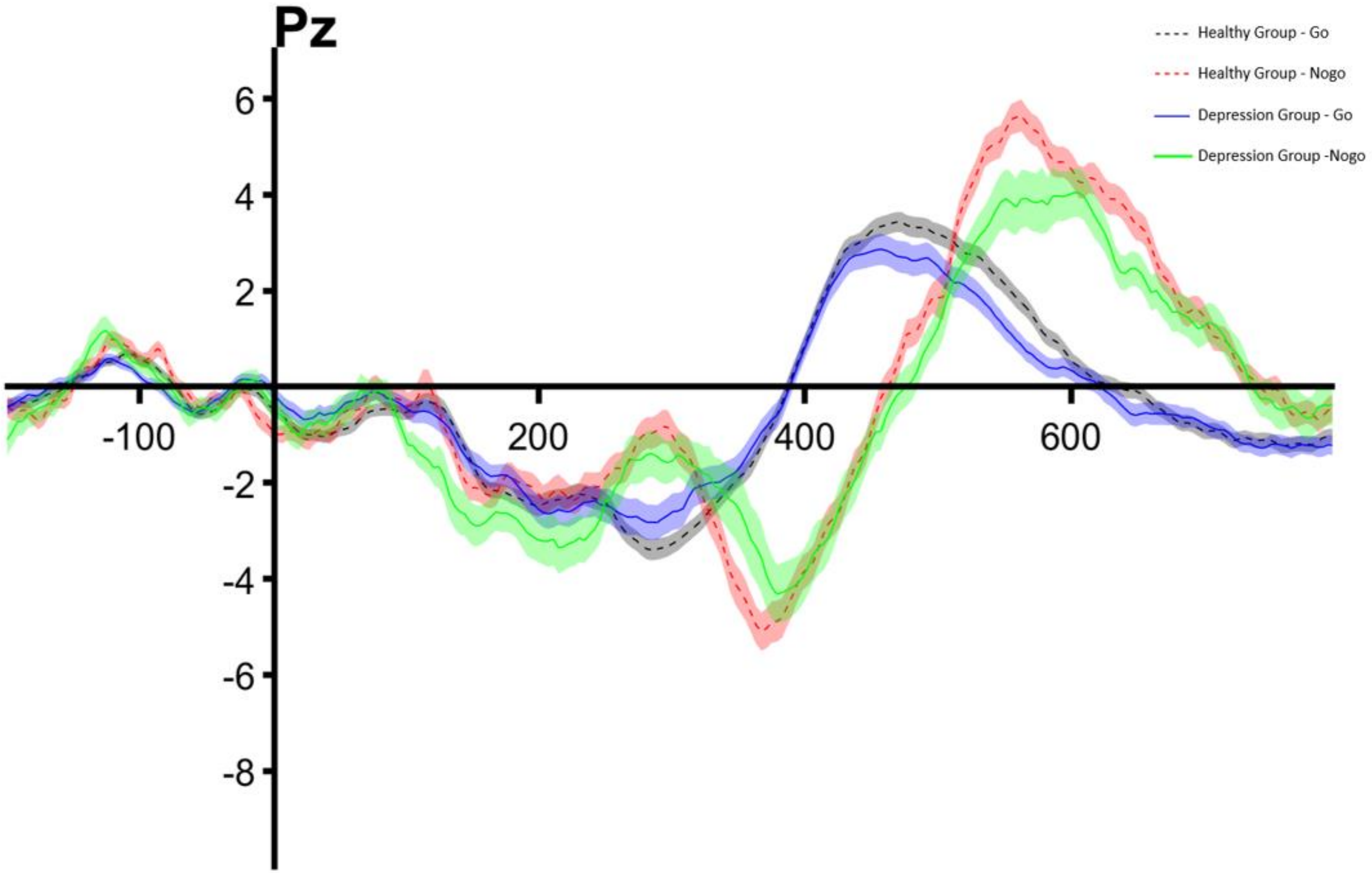

Figure 3 Pz potentials induced by the Go/No-Go task

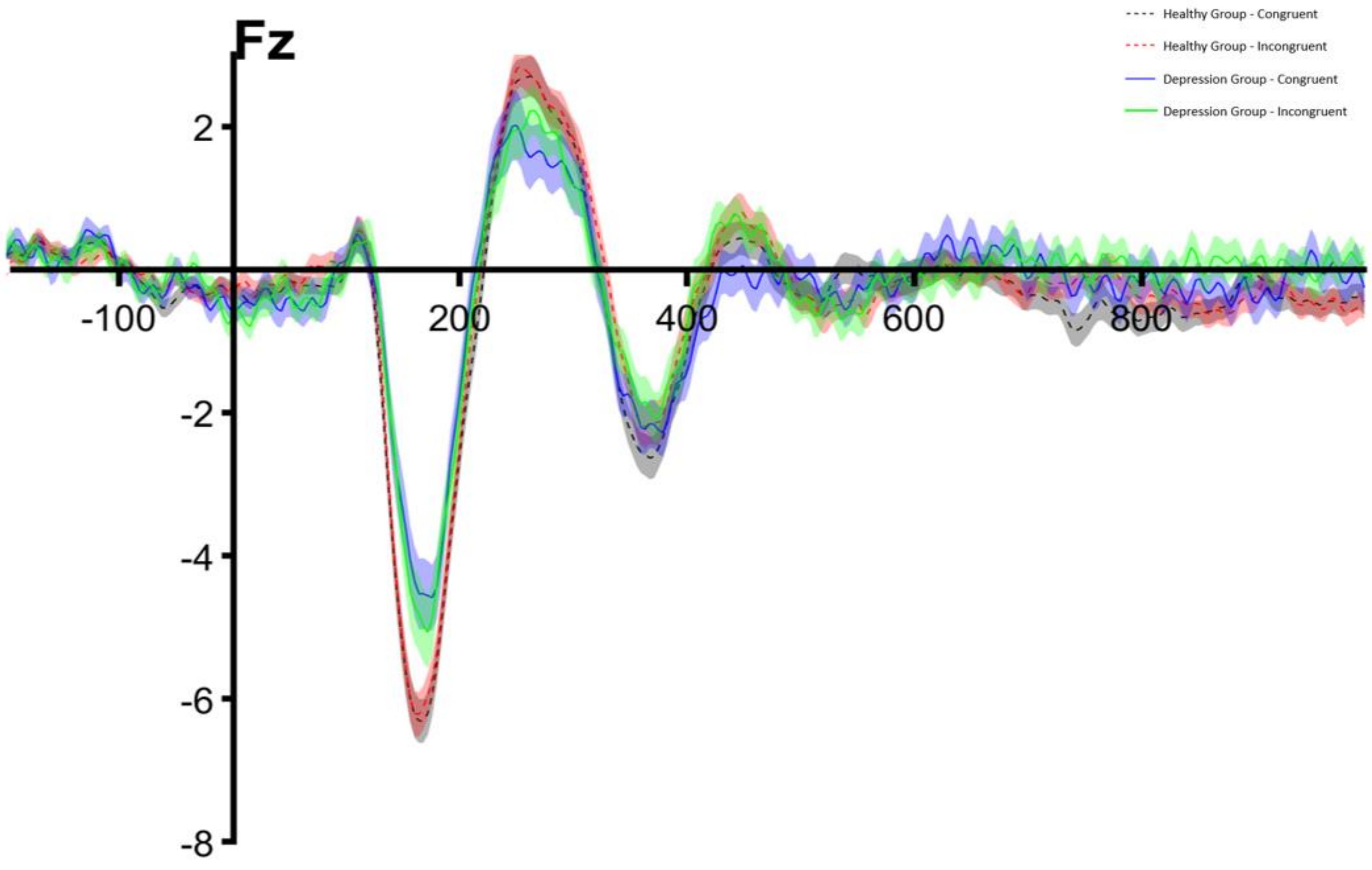

Figure 3 Fz potentials induced by the Stroop task

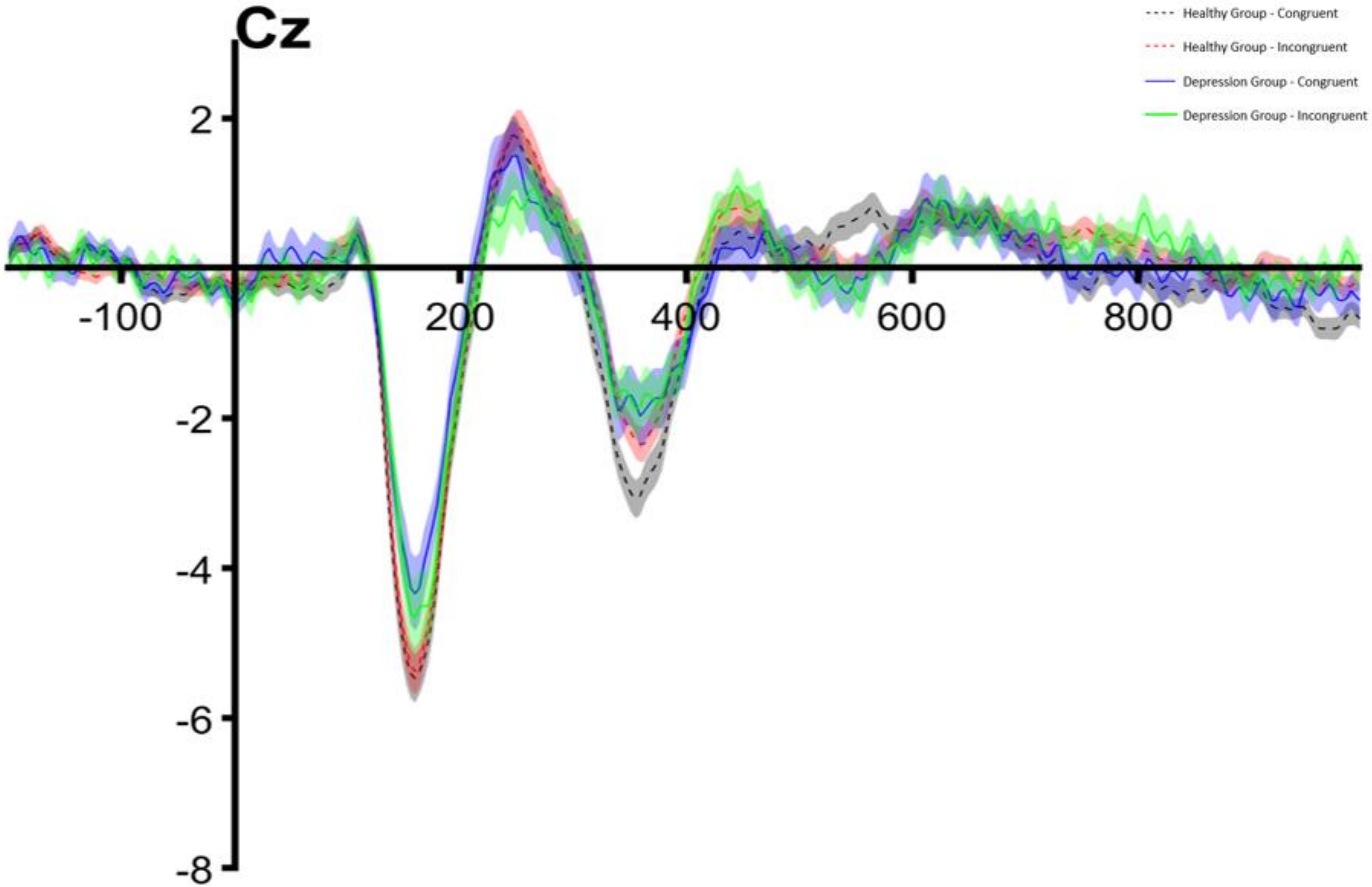

Figure 3 Cz potentials induced by the Stroop task

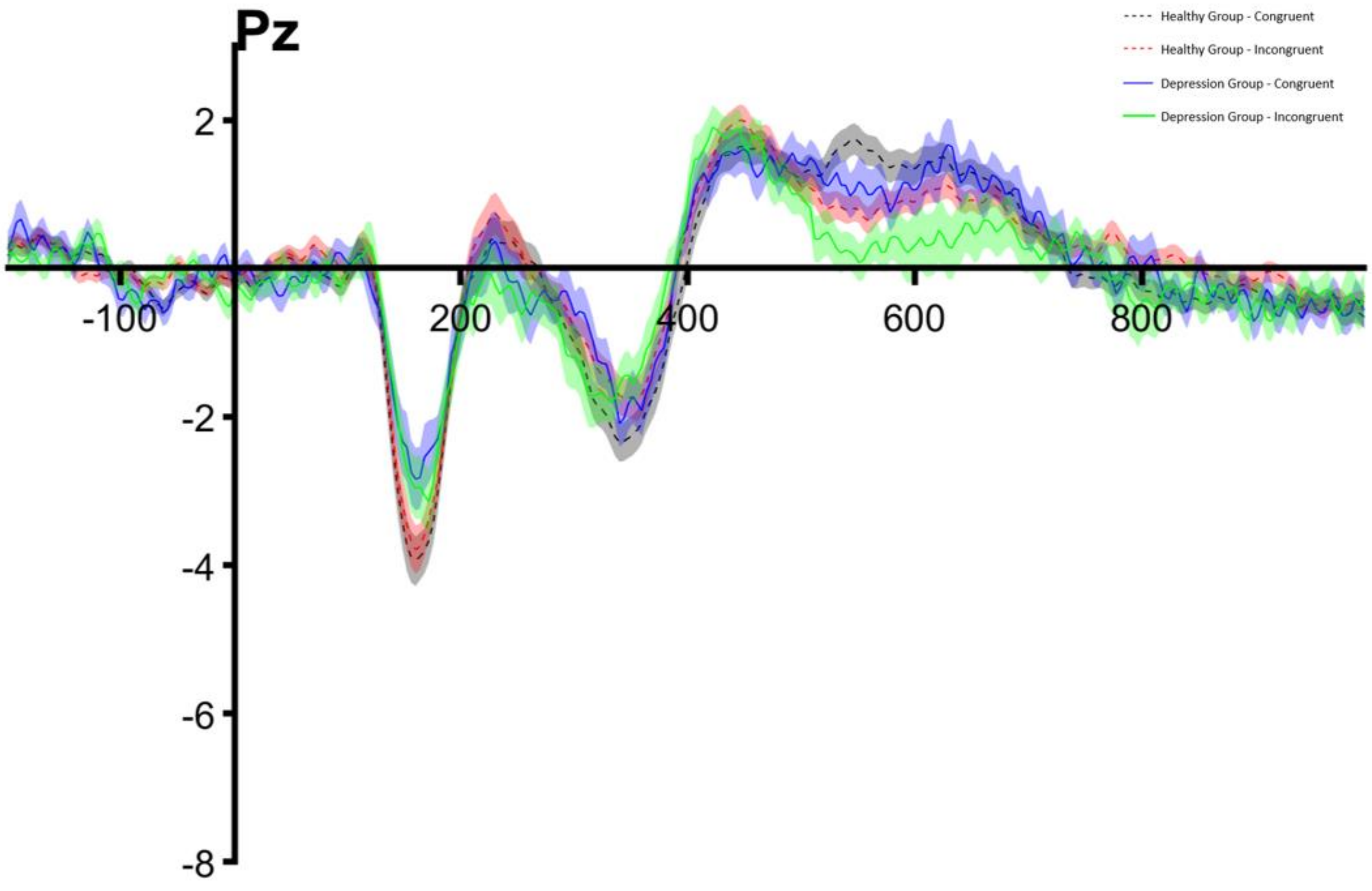

Figure 3 Pz potentials induced by the Stroop task

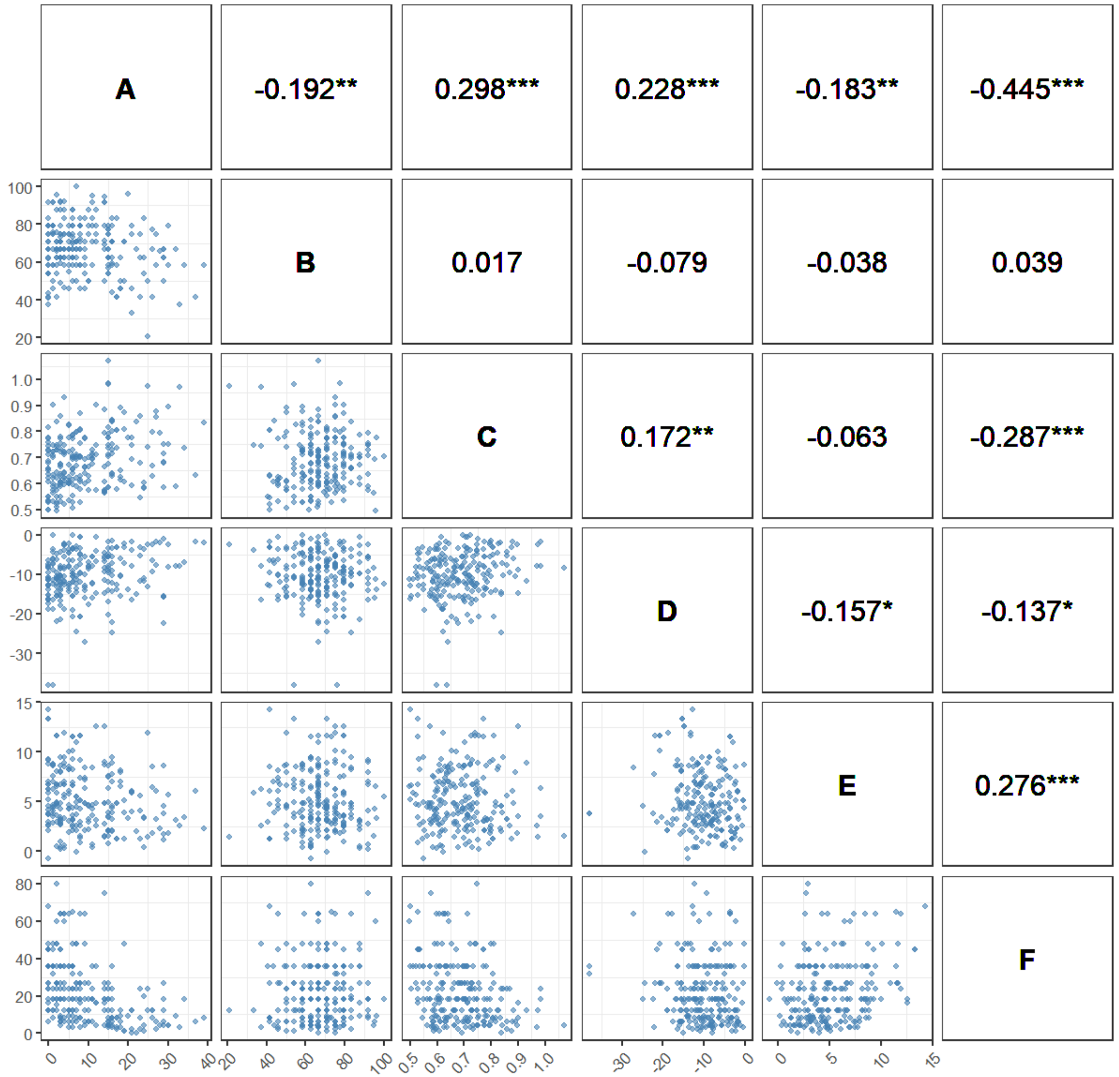

Figure 4. (1) Relationships between Physical Exercise Volume, Depressive Symptom Scores, and Inhibitory Function-Specific Indicators.

Note: *$p < 0.05$, **$p < 0.01$, ***$p < 0.001$. A represents depressive symptom scores; B represents Nogo accuracy; C represents Stroop incongruent reaction time; D represents the N2 amplitude of Fz potentials under the Nogo condition; E represents the P3 amplitude of Fz potentials under the Stroop incongruent condition; F represents physical exercise volume.

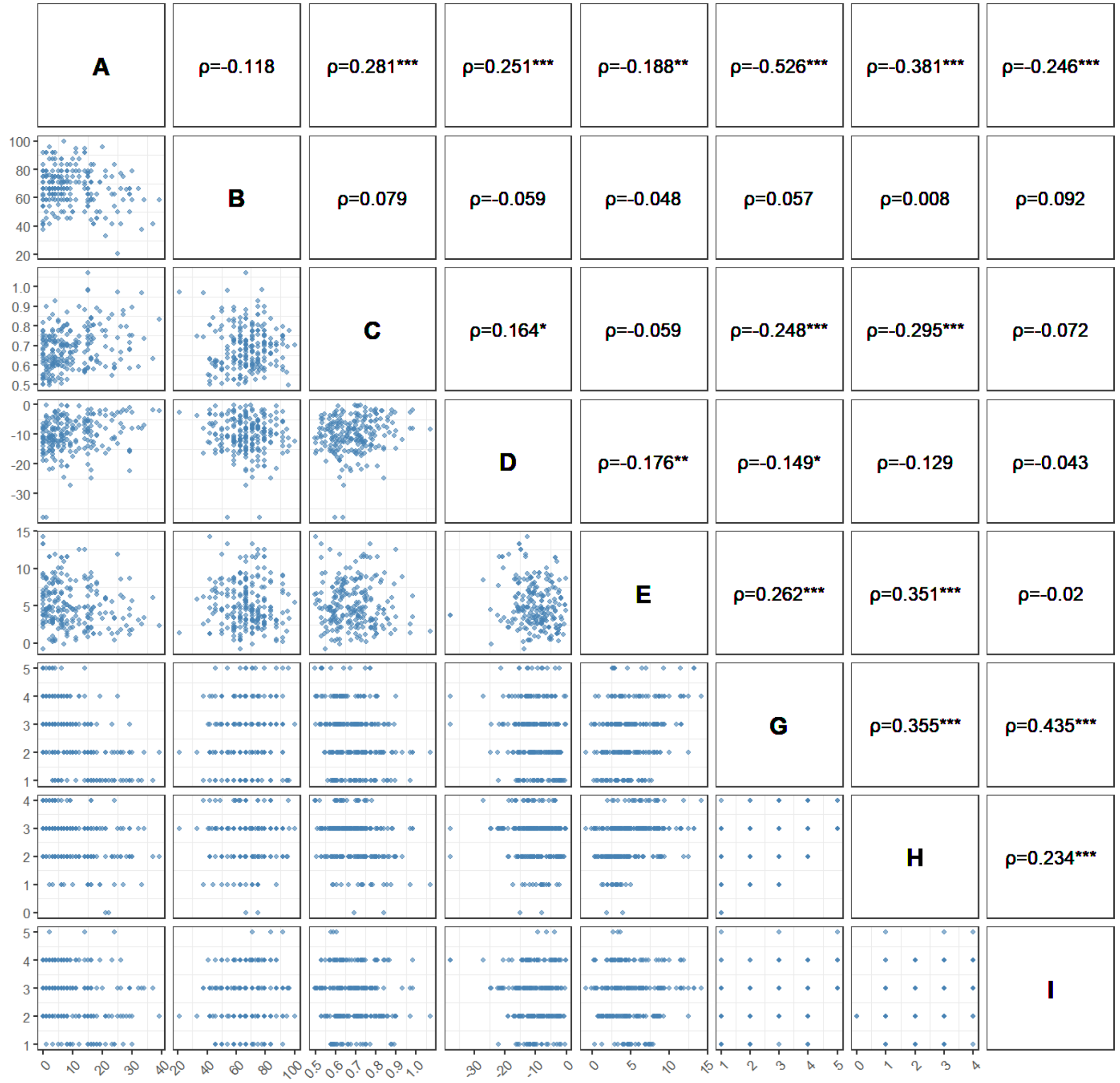

Figure 4. (2)Relationships between Physical Exercise Intensity, Duration, Frequency, Depressive Symptom Scores, and Inhibitory Function-Specific Indicators.

Note: *$p < 0.05$, **$p < 0.01$, ***$p < 0.001$. A represents depressive symptom scores; B represents Nogo accuracy; C represents Stroop incongruent reaction time; D represents the N2 amplitude of Fz potentials under the Nogo condition; E represents the P3 amplitude of Fz potentials under the Stroop incongruent condition; G represents intensity; H represents duration; I represents frequency.
